# *SEMA7A*^R148W^ mutation promotes lipid accumulation and NAFLD progression via increased localization on the hepatocyte surface

**DOI:** 10.1172/jci.insight.154113

**Published:** 2022-08-08

**Authors:** Nan Zhao, Xiaoxun Zhang, Jingjing Ding, Qiong Pan, Ming-Hua Zheng, Wen-Yue Liu, Gang Luo, Jiaquan Qu, Mingqiao Li, Ling Li, Ying Cheng, Ying Peng, Qiaoling Xie, Qinglin Wei, Qiao Li, Lingyun Zou, Xinshou Ouyang, Shi-Ying Cai, James L. Boyer, Jin Chai

**Affiliations:** 1 Department of Gastroenterology, Institute of Digestive Diseases of PLA, Cholestatic Liver Diseases Center, and Center for Metabolic Associated Fatty Liver Disease, The First Affiliated Hospital (Southwest Hospital) of Third Military Medical University (Army Medical University), Chongqing, China.; 2 NAFLD Research Center, Department of Hepatology, and; 3 Department of Endocrinology, The First Affiliated Hospital of Wenzhou Medical University, Wenzhou, China.; 4 Bioinformatics Center, Department of Microbiology of Third Military Medical University, Chongqing, China.; 5 Bao’an Maternal and Child Health Hospital of Jinan University, Shenzhen, China.; 6 Department of Internal Medicine, Section of Digestive Diseases, and; 7 Department of Internal Medicine and Liver Center, Yale University School of Medicine, New Haven, Connecticut, USA.

**Keywords:** Hepatology, Metabolism, Genetic variation, Molecular biology, Mouse models

## Abstract

Genetic polymorphisms are associated with the development of nonalcoholic fatty liver disease (NAFLD). *Semaphorin7a* (*Sema7a*) deficiency in mouse peritoneal macrophages reduces fatty acid (FA) oxidation. Here, we identified 17 individuals with *SEMA7A* heterozygous mutations in 470 patients with biopsy-proven NAFLD. *SEMA7A* heterozygous mutations increased susceptibility to NAFLD, steatosis severity, and NAFLD activity scores in humans and mice. The *Sema7a*^R145W^ mutation (equivalent to human *SEMA7A*^R148W^) significantly induced small lipid droplet accumulation in mouse livers compared with WT mouse livers. Mechanistically, the *Sema7a*^R145W^ mutation increased N-glycosylated Sema7a and its receptor integrin β1 proteins in the cell membranes of hepatocytes. Furthermore, *Sema7a*^R145W^ mutation enhanced its protein interaction with integrin β1 and PKC-α and increased PKC-α phosphorylation, which were both abrogated by integrin β1 silencing. Induction of *PKCα*_WT, but not *PKCα*_dominant negative, overexpression induced transcriptional factors Srebp1, Chrebp, and Lxr expression and their downstream Acc1, Fasn, and Cd36 expression in primary mouse hepatocytes. Collectively, our findings demonstrate that the *SEMA7A*^R148W^ mutation is a potentially new strong genetic determinant of NAFLD and promotes intrahepatic lipid accumulation and NAFLD in mice by enhancing PKC-α–stimulated FA and triglyceride synthesis and FA uptake. The inhibition of hepatic PKC-α signaling may lead to novel NAFLD therapies.

## Introduction

Nonalcoholic fatty liver disease (NAFLD) is a common health issue, and its prevalence is rapidly increasing worldwide. NAFLD and its more advanced form, nonalcoholic steatohepatitis (NASH), have the potential to progress into cirrhosis and hepatocellular carcinoma (1–3). NAFLD is characterized by excessive accumulation of lipids in hepatocytes leading to chronic inflammation and liver damage in patients (1–3). The liver is the most important organ for metabolizing fatty acids (FAs) and triglycerides (TGs) as well as maintaining lipid homeostasis. The AMP kinase (AMPK) and protein kinase C (PKC) signaling pathways are mainly responsible for regulating these physiological functions (1). Liver dysregulation can promote the accumulation of excess lipids within, leading to hepatic steatosis and NAFLD (1). It is well known that multiple factors, including genetic and environmental factors, contribute to the development of NAFLD (1–3). However, the mechanisms underlying the pathological process of NAFLD remain to be elucidated. At present, several genetic risk factors for NAFLD have been identified, such as polymorphisms of patatin like phospholipase domain containing 3 (*PNPLA3*), transmembrane 6 superfamily member 2, and hydroxysteroid 17-beta dehydrogenase 13 (2). The polymorphism (rs738409, c.444C>G) of *PNPLA3*, encoding an adiponutrin protein associated with lipid droplets, is a strong genetic determinant of NAFLD (2). However, whether additional genetic factors are also important for the development of NAFLD remains to be determined.

Semaphorins are extracellular signaling proteins and can bind to their membrane receptors of plexins and integrins (4). They are essential for the development of many organs and tissues and the maintenance of their functions (4). Semaphorin 7A (SEMA7A), known as the John Milton Hagen antigen or CD108, is a glycosylphosphatidylinositol-anchored membrane protein with chemoattractant and chemorepulsive attributes (5). SEMA7A is expressed in multiple tissues, including the liver (4–7). Functionally, SEMA7A is crucial for axon growth, T cell activation, and other biological processes by binding to its receptors of integrin β1 and plexin C1 (4–9). Mutations in *SEMA7A* are associated with decreased bone mineral density and Kallmann syndrome (10, 11). Recently, Körner et al. (12) reported that *Sema7a* deficiency in mouse peritoneal macrophages reduced FA oxidation and oxidative phosphorylation, suggesting that SEMA7A may regulate lipid metabolism. Our preliminary study characterized mice with a missense mutation in *Sema7a*, and we found that these mice developed hydropic and fatty degeneration in hepatocytes. Therefore, we speculate that *SEMA7A* mutations may contribute to lipid metabolic disorders and NAFLD development.

In this study, we identified 17 patients with *SEMA7A* heterozygous mutation in 470 patients with biopsy-proven NAFLD. Remarkably, *SEMA7A* heterozygous mutations increased susceptibility to and severity of NAFLD in human patients and mouse models. The *Sema7a*^R145W^ mutation caused intrahepatic accumulation of small lipid droplets in mice by activating PKC-α signaling. In our study, we provide what we believe is new evidence that the *SEMA7*A^R148W^ mutation is a new genetic determinant of NAFLD and uncover the molecular mechanisms underlying the role of *SEMA7A*^R148W^ mutation in the development of NAFLD.

## Results

### SEMA7A heterozygous mutations are potentially novel risk factors for human NAFLD.

To examine whether SEMA7A mutations contribute to the development of NAFLD in humans, we performed exon sequencing of *SEMA7A* (Supplemental Table 1; supplemental material available online with this article; https://doi.org/10.1172/jci.insight.154113DS1) in 470 patients with biopsy-proven NAFLD (13). We identified 17 NAFLD patients with *SEMA7A* heterozygous mutations, including p.R148W (*n* = 5), p.V334I (*n* = 4), p.R302K (*n* = 3), p.T2M (*n* = 2), p.P74L, p.R66Q, and p.N559Y (*n* = 1 each) (Table 1). There was no patient with in-frame deletion, insertion, or frameshift in exons of *SEMA7A*. Strikingly, the frequency of candidate variants in *SEMA7A* in the NAFLD cohort (3.62%; 17 variants in 470 patients with NAFLD) was significantly higher than that of East Asian individuals (0.29%; 54 variants in 18,394 individuals) in the gnomAD, leading to an OR of 12.75 (95% CI: 7.33–22.16; *P* < 0.00001) (Table 1). These findings indicated that *SEMA7A* heterozygous mutations were risk factors for the development of NAFLD in humans.

### SEMA7A heterozygous mutations are significantly associated with human NAFLD severity.

Next, we analyzed livers from 13 out of 17 patients with *SEMA7A* heterozygous mutations and their pair-matched controls from these 470 NAFLD patients, excluding the 4 patients without pair-matched controls (Table 2). Liver histological analysis showed that the severity of steatosis and NASs were significantly higher in NAFLD patients with *SEMA7A* heterozygous mutations than those of their matched controls (*P* = 0.032 and *P* = 0.012, respectively) (Table 2 and Supplemental Figure 1). Moreover, serum ALT, AST, and GGT levels in NAFLD patients with *SEMA7A* heterozygous mutations were also higher than in the paired controls (*P* = 0.023, *P* = 0.100, and *P* = 0.345, respectively) (Table 2). However, there were no significant differences in hepatocyte ballooning, lobular inflammation, and fibrosis between these groups (Table 2). Taken together, *SEMA7A* heterozygous mutations in this population were associated with increased severity of NAFLD.

### The Sema7a^R145W^ heterozygous mutation markedly increases NAFLD severity and liver enzyme levels in mice following high-fat diet.

**Figure 1:**
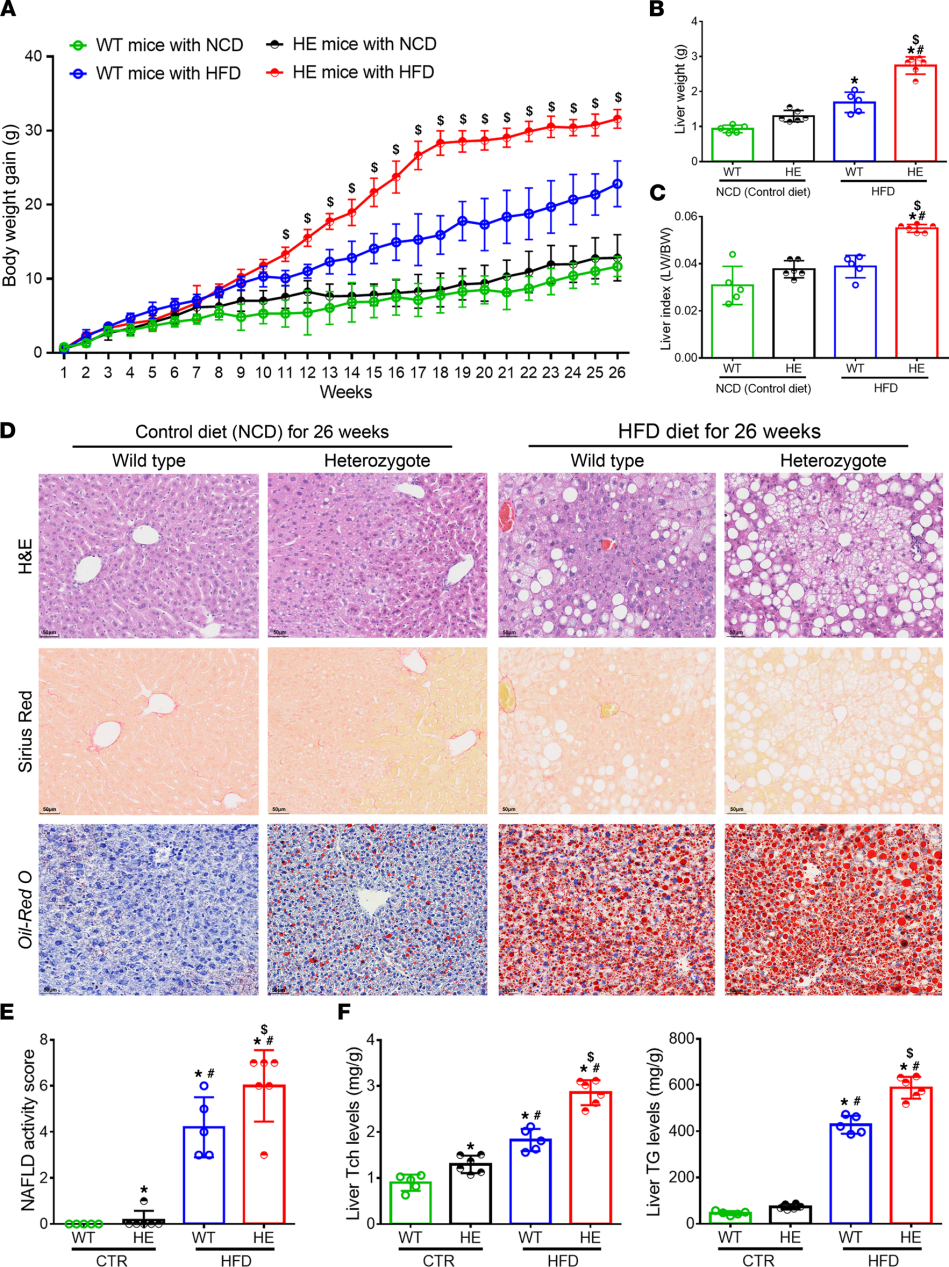
The *Sema7a*^R145W^ heterozygous mutation promotes the progression of NAFLD in mice following HFD feeding. Male WT and *Sema7a*^R145W^ heterozygous (HE) mice were fed with normal chow diet (NCD) or HFD for 26 weeks. (**A**) The dynamic changes in the gains of body weights. (**B**) Liver weights. (**C**) The liver/body weight ratios. NCD-WT (*n* = 5), NCD-HE (*n* = 6), HFD-WT (*n* = 5), HFD-HE (*n* = 6). (**D**) Representative images (original magnification, ×200) of H&E, Oil Red O, and Sirius red staining of liver sections. (**E**) Quantitative analysis of the NAFLD activity scores (NASs). (**F**) Hepatic TG and Tch levels. The data were analyzed by 1-way ANOVA with Tukey’s post hoc tests or by Kruskal-Wallis test with Dunn’s post hoc test analysis. *P < 0.05 versus the WT mice with NCD; ^#^*P* < 0.05 versus the *Sema7a*^R145W^ heterozygous mice with NCD; ^$^*P* < 0.05 versus the WT mice with HFD. TG, triglycerides; Tch, total cholesterol.

Single-cell RNA sequencing of *SEMA7A* in human liver cells revealed that *SEMA7A* was expressed by hepatocytes as well as all other types of liver cells (Supplemental Figure 2). Because 5 out of the 13 NAFLD patients with *SEMA7A* mutations were heterozygous for *SEMA7A*^R148W^, we also characterized *Sema7a*^R145W^ (equivalent to human *SEMA7A*^R148W^) heterozygous mice in order to further investigate the functional role of *SEMA7A* mutations in the progression of NAFLD. Following feeding with high-fat diet (HFD) for 26 weeks, we found that the body weight gains, liver weights, and liver/body weight ratios in the *Sema7a*^R145W^ heterozygotes were significantly higher than those of WT controls (Figure 1, A–C). Histological assessments revealed that hepatic lipid droplets and NASs in the HFD-fed *Sema7a*^R145W^ heterozygous mice were also significantly increased compared with the HFD-fed WT mice (Figure 1, D and E). In comparison with the WT controls, the levels of hepatic TG, total cholesterol (Tch), and serum ALT and AST were also significantly increased (Figure 1F and Supplemental Table 2). Thus, the *Sema7a*^R145W^ heterozygous mutation promoted the progression of NAFLD in mice.

### The Sema7a^R145W^ mutation significantly increases the accumulation of small lipid droplets in mouse livers.

**Figure 2:**
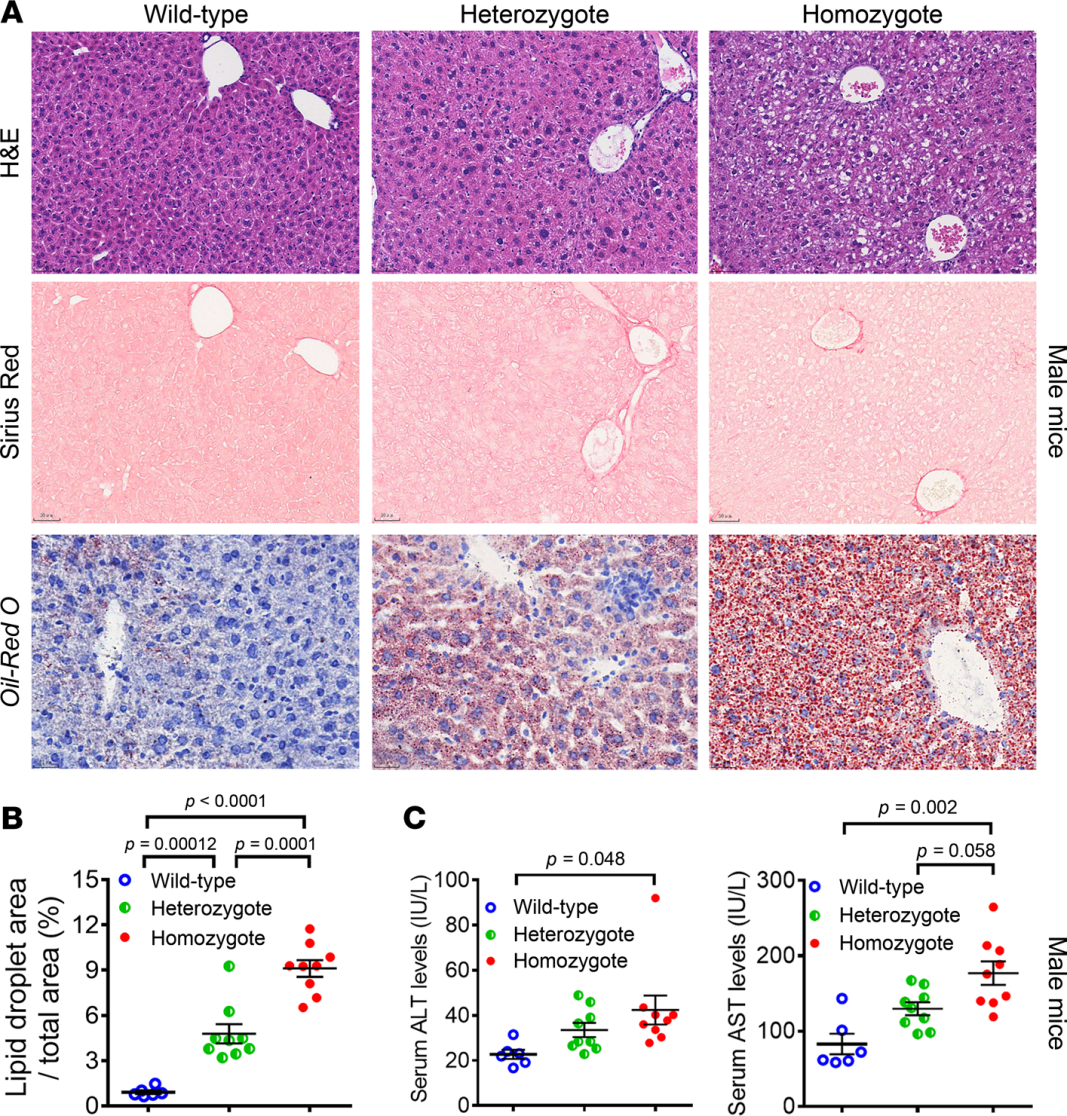
The *Sema7a*^R145W^ mutation causes intrahepatic accumulation of small lipid droplets in male mice at 10 weeks of age. (**A**) Representative images (original magnification, ×200) of H&E staining, Sirius red staining, and Oil Red O staining in WT and *Sema7a*^R145W^ heterozygous and homozygous male mice at the age of 10 weeks. (**B**) Analysis of lipid droplets in the Oil Red O–stained liver sections of WT (*n* = 6) and *Sema7a*^R145W^ heterozygous (*n* = 9) and homozygous male mice (*n* = 9). (**C**) The levels of serum ALT and AST in *Sema7a*^R145W^ WT (*n* = 6), heterozygous (*n* = 9), and homozygous male mice (*n* = 9). The data were analyzed by 1-way ANOVA with Tukey’s post hoc tests or by Kruskal-Wallis test with Dunn’s post hoc test analysis.

Oil Red O staining displayed the markedly increased accumulation of small lipid droplets in the liver sections of *Sema7a*^R145W^ homozygous mice at 10 weeks of age, compared with the age-matched WT and heterozygous mice (Figure 2, A and B; and Supplemental Figure 3, A and B). Moreover, the areas of hepatic small lipid droplets in heterozygous mice were also significantly larger than in the WT control mice (Figure 2, A and B; and Supplemental Figure 3, A and B). Furthermore, *Sema7a*^R145W^ homozygous mice showed levels of serum ALT and AST that were significantly greater than WT and heterozygous mice, although there was no significant difference in these measurements between heterozygotes and WT controls (Figure 2C and Supplemental Figure 3C). Taken together, the *Sema7a*^R145W^ mutation induced intrahepatic accumulation of small lipid droplets and enhanced liver injury in mice.

### The Sema7a^R145W^ mutation enhances PKC-α activation to stimulate FA and TG synthesis and FA uptake in mouse livers.

**Figure 3:**
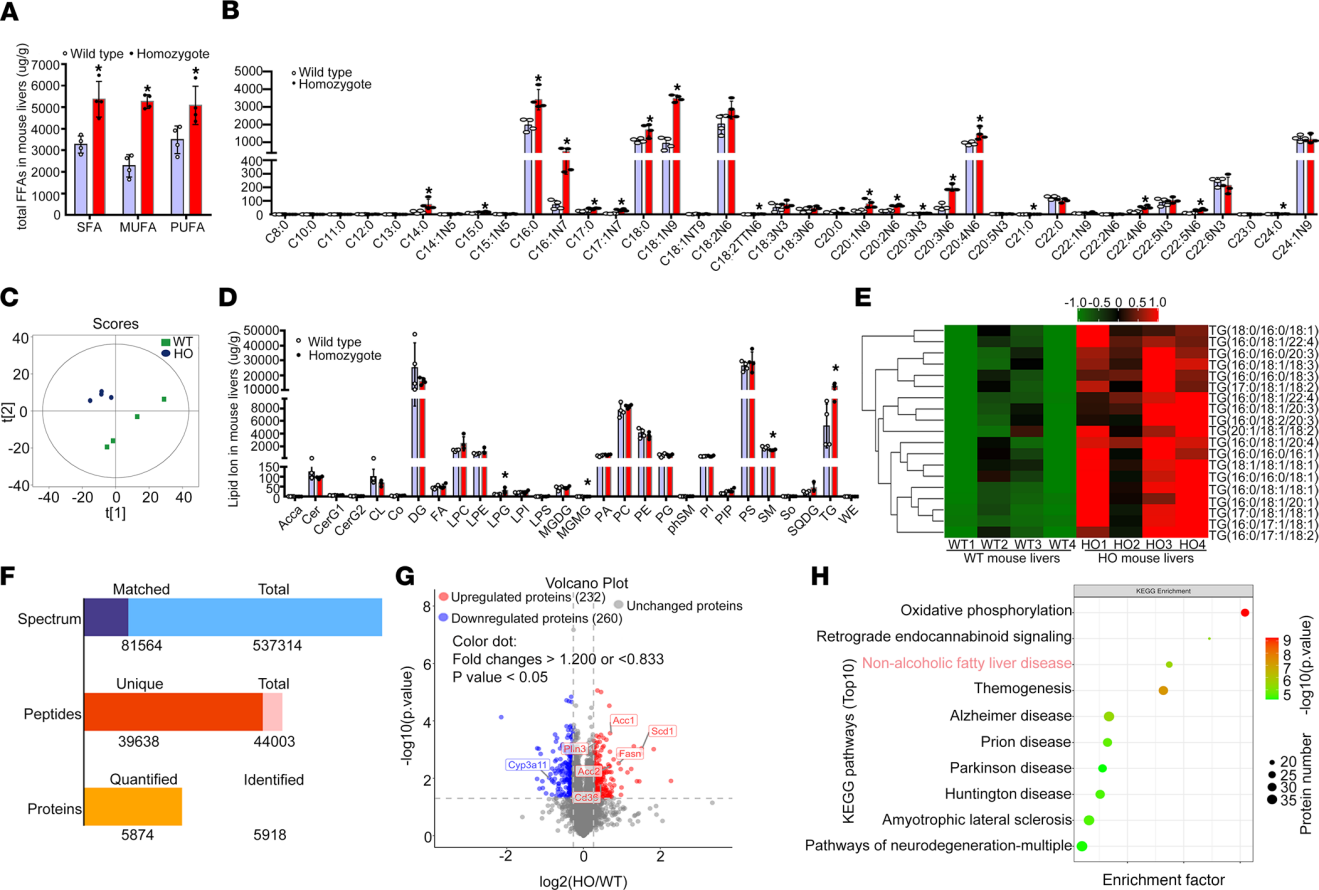
The *Sema7a*^R145W^ mutation increases hepatic FA and TG concentrations in mouse livers. Male WT and *Sema7a*^R145W^ homozygous (HO) mice at 10 weeks old (*n* = 4 per group) were euthanized and their liver samples were prepared. (**A**) Gas chromatography tandem mass spectrometry (GC/MS) analysis of total saturated fatty acid (SFA), monounsaturated fatty acid (MUFA), and polyunsaturated fatty acid (PUFA) levels (μg/g of mouse liver) in WT and *Sema7a*^R145W^ homozygous mouse livers. FFA, free fatty acid. (**B**) Quantification of hepatic long chain FA (FA μg/g of mouse liver) in WT and *Sema7a*^R145W^ homozygous mice. (**C**) Score scatterplot corresponding to a principal component analysis of the lipidomic data in the livers of WT and *Sema7a*^R145W^ homozygous mice. (**D**) Quantitative analysis of lipid ion (μg/g of mouse liver) in WT and *Sema7a*^R145W^ homozygous mouse livers. (**E**) Heatmap analysis of the TG number of carbons and double bond contents in the livers of WT and *Sema7a*^R145W^ homozygous mice. (**F**) Proteomic analysis in the livers of WT and *Sema7a*^R145W^ heterozygous and homozygous mice (*n* = 5 per group). (**G**) Volcano plot of the quantified proteins from WT and *Sema7a*^R145W^ homozygous mouse livers (*n* = 5 per group). (**H**) Kyoto Encyclopedia of Genes and Genomes (KEGG) analysis of the differentially expressed genes in the pathways between WT and *Sema7a*^R145W^ homozygous mice. The data were analyzed by independent-sample *t* test, 2-tailed. *P < 0.05 versus the WT mice.

**Figure 4:**
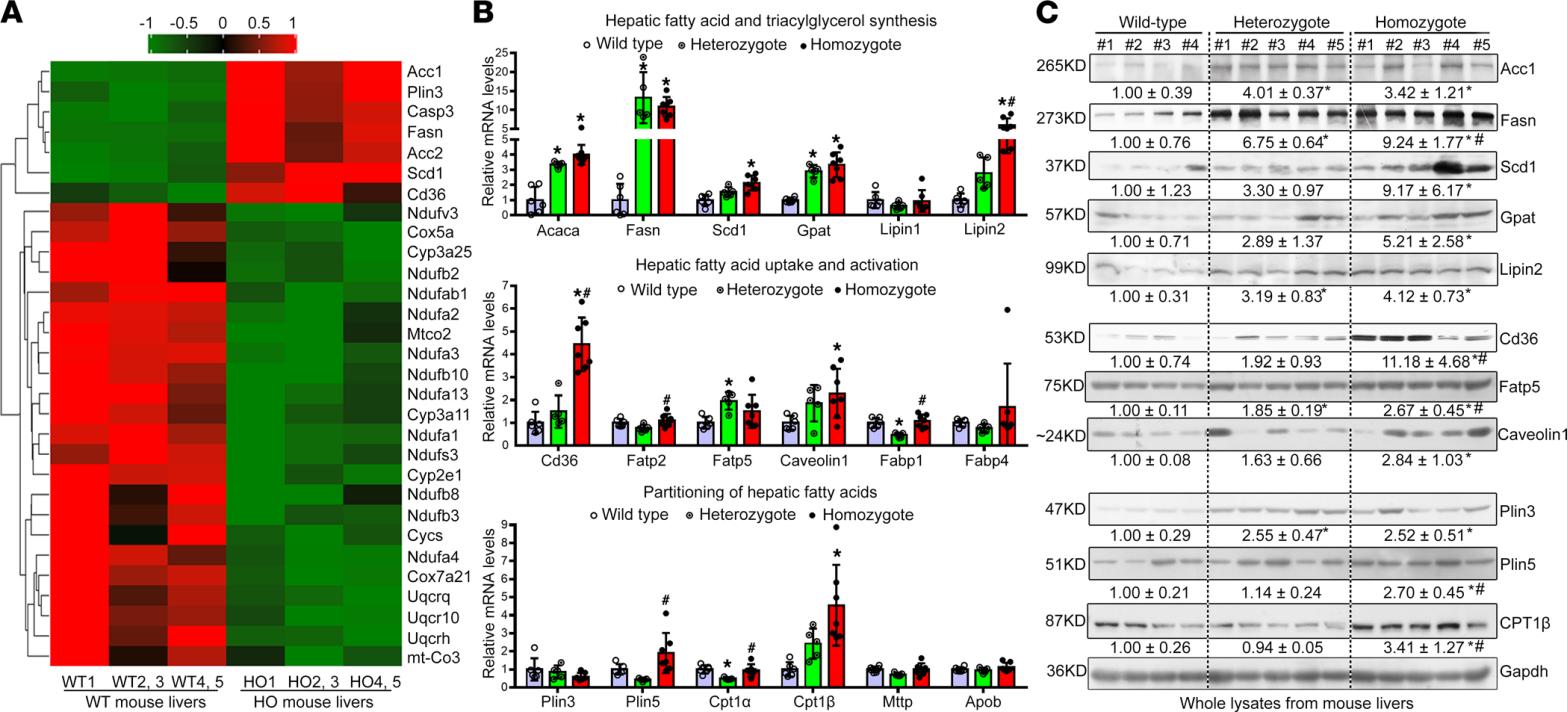
The *Sema7a*^R145W^ mutation increases FA and TG synthesis and FA uptake in mouse livers. (**A**) Proteomic heatmap analysis of the NAFLD pathway in the livers of WT and *Sema7a*^R145W^ homozygous mice. The liver extracts from the WT2 and 3, WT4 and 5, HO2 and 3, and HO4 and 5 male mice were combined for proteomics analysis. (**B**) The relative levels of mRNA transcripts of the genes for FA and TG synthetic enzymes, FA uptake transporters, and FA partitioning in WT mice (*n* = 6), *Sema7a*^R145W^ heterozygous mice (*n* = 5), and *Sema7a*^R145W^ homozygous mice (*n* = 7). (**C**) Western blot analysis of the relative levels of Acc1, Fasn, Scd1, Gpat, Lipin2, Cd36, Fatp5, Caveolin1, Plin3, Plin5, and Cpt1β protein expression in the livers of WT (*n* = 4), *Sema7a*^R145W^ heterozygous (*n* = 5), and *Sema7a*^R145W^ homozygous (*n* = 5) mice. The data were analyzed by 1-way ANOVA with Tukey’s post hoc tests or by Kruskal-Wallis test with Dunn’s post hoc test analysis. *P < 0.05 versus the WT mice, ^#^*P* < 0.05 versus the *Sema7a*^R145W^ heterozygous mice.

Targeted metabolomics of FA and quantitative lipidomic analyses indicated that the *Sema7a*^R145W^ homozygous mutation remarkably increased hepatic FA and TG concentrations in mice, relative to the controls (Figure 3, A–E, and Supplemental Tables 3–5). Next, proteomics analysis revealed that, among 5874 quantified proteins, 232 were upregulated and 260 were downregulated (Figure 3, F and G). Kyoto Encyclopedia of Genes and Genomes (KEGG) analysis exhibited that the NAFLD pathway (in the top 3) was enriched in *Sema7a*^R145W^ homozygous mouse livers (Figure 3H and Supplemental Table 6). Proteomics heatmap, real-time quantitative PCR (qPCR), and Western blotting analyses revealed that the *Sema7a*^R145W^ homozygous mutation increased the expression of key genes for hepatic FA and TG synthesis (Acaca/Acc1, Fasn, Scd1, Gpat, and Lipin2), FA uptake (Cd36, Fatp5, and Caveolin1), and FA partitioning (Plin3, Plin5, and Cpt1β) (Figure 4, A–C). Similar patterns of moderate alterations were detected in heterozygous mice, compared to the WT controls (Figure 4, A–C). These data indicated that the *Sema7a*^R145W^ mutation stimulated hepatic FA and TG synthesis and FA uptake in mice.

**Figure 5:**
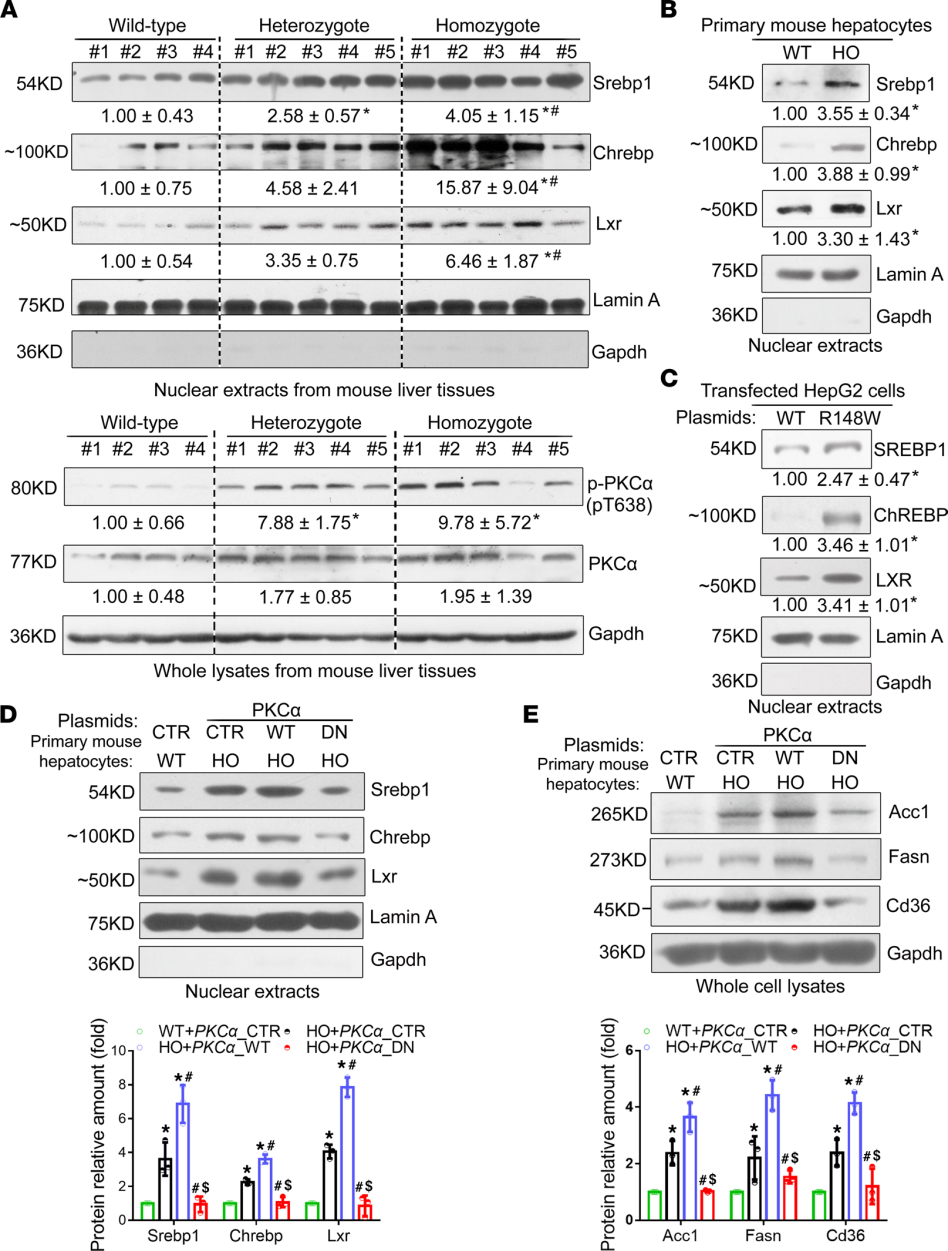
The *Sema7a*^R145W^ mutation enhances hepatic FA and TG synthesis and FA uptake by enhancing PKC-α signaling–stimulated expression of transcription factors Srebp1 and Chrebp and nuclear receptor Lxr. (**A**) Western blot analysis of the relative levels of Srebp1, Chrebp, Lxr, phosphorylated PKC-α, and PKC-α protein expression in 10-week-old male WT mice (*n* = 4), *Sema7a*^R145W^ heterozygous mice (*n* = 5), and *Sema7a*^R145W^ homozygous mice (*n* = 5). Western blot analysis of the relative levels of nuclear Srebp1, Chrebp, and Lxr proteins in primary hepatocytes from WT and *Sema7a*^R145W^ homozygous mice (**B**) and in human hepatoma HepG2 cells (**C**) after transfection with the plasmid for the expression of *SEMA7A*_WT and *SEMA7A*_R148W proteins. (**D**) Representative Western blot of the relative levels of nuclear Srebp1, Chrebp, and Lxr proteins in nuclear extracts and (**E**) Fasn, Acc1, and Cd36 proteins in whole-cell lysates of primary mouse hepatocytes after transfection with empty vector (CTR) or the plasmid for the expression of *PKCα*_WT or *PKCα*_dominant negative (DN) mutant, respectively. All primary mouse hepatocytes were isolated from 12-week-old male WT and *Sema7a*^R145W^ homozygous mice. Data are representative images or expressed as the mean ± SD of each group from 3 separate experiments. The data were analyzed by 1-way ANOVA with Tukey’s post hoc tests or by Kruskal-Wallis test with Dunn’s post hoc test analysis. *P < 0.05 versus the WT mice, ^#^*P* < 0.05 versus the *Sema7a*^R145W^ heterozygous mice, ^$^*P* < 0.05 versus the primary HO mouse hepatocytes transfected with CTR; *n* = 3.

A recent study reported that PKC-α signaling mediates lipid metabolism in diabetic rats (14). Our further analyses of hepatic expression of nuclear Srebp1, Chrebp, Lxr and their targeting genes of Acc1, Fasn, Scd1, Cd36, along with PKC-α phosphorylation, demonstrated that their expression levels were significantly higher in the liver and primary hepatocytes from *Sema7a*^R145W^ homozygous mice than that from the WT controls (Figure 4, B and C; and Figure 5, A and B). Similar results were obtained in NAFLD patients with *SEMA7A* mutation, *Sema7a*^R145W^ heterozygous mouse livers, and HepG2 cells transfected with *SEMA7A*_R148W construct (Figure 5, A and C, and Supplemental Figure 4). However, there was no significant difference in the relative levels of AMPK phosphorylation among these groups of mice (data not shown). These data indicated that the *Sema7a*^R145W^ mutation increased PKC-α phosphorylation and nuclear Srebp1, Chrebp, and Lxr expression in the livers of mice. Furthermore, induction of *PKCα*_WT, but not its dominant negative (*PKCα*_DN), overexpression markedly increased nuclear Srebp1, Chrebp, and Lxr expression and their downstream Acc1, Fasn, and Cd36 expression in primary *Sema7a*^R145W^ homozygous mouse hepatocytes (Figure 5, D and E). These findings indicate that the *Sema7a*^R145W^ mutation enhanced FA and TG synthesis and FA uptake by increasing PKC-α–stimulated nuclear Srebp1, Chrebp, and Lxr expression in hepatocytes of mice.

### The Sema7a^R145W^ mutation increases its protein in cell surface membranes and activates the PKC-α signaling in hepatocytes.

**Figure 6:**
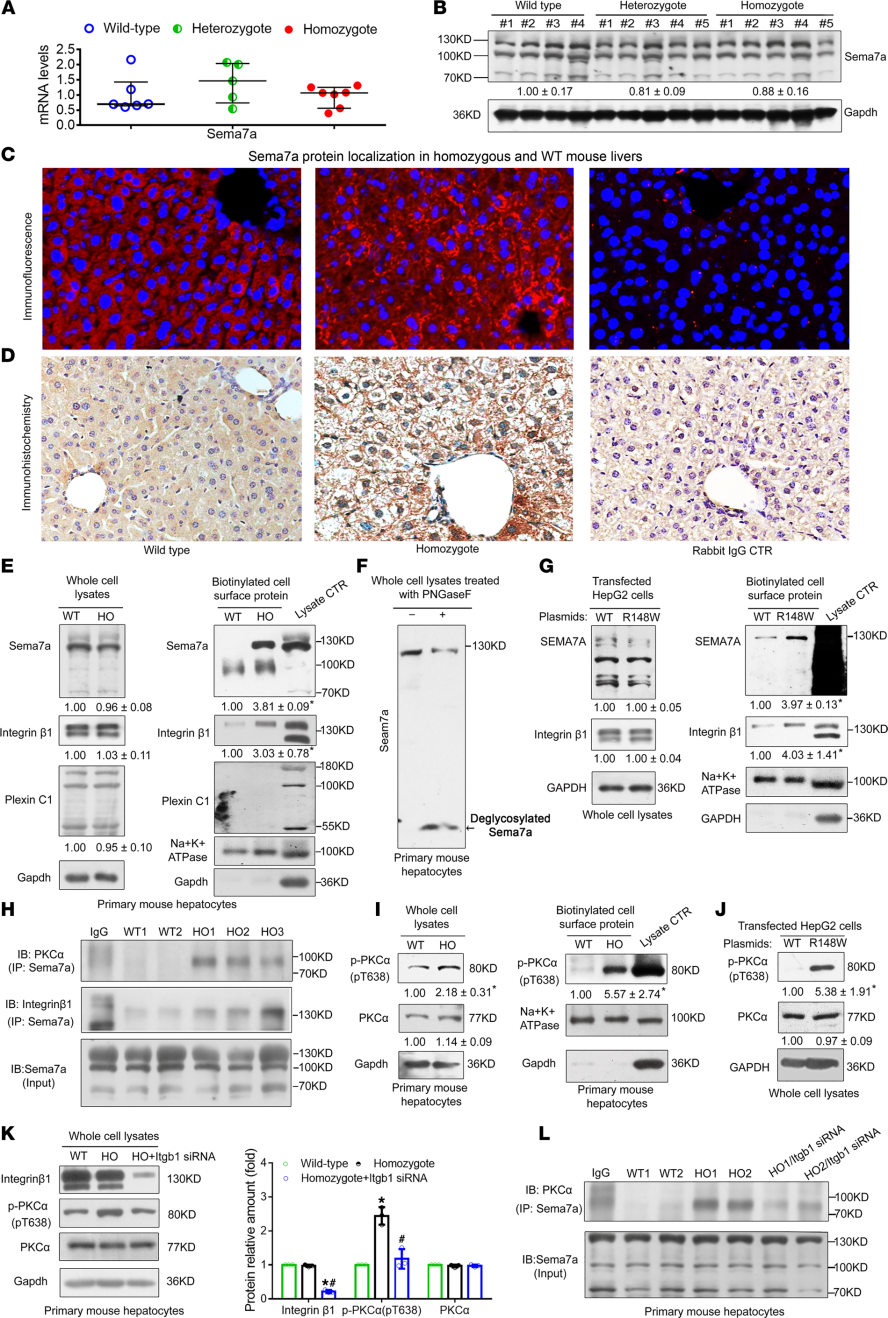
The *Sema7a*^R145W^ mutation does not alter total Sema7a expression but increases Sema7a and integrin β1 in the cell surface membrane and activates PKC-α signaling in hepatocytes. Relative levels of Sema7a mRNA transcripts (**A**) and protein expression (**B**) in 10-week-old male WT, *Sema7a*^R145W^ heterozygous, and homozygous mice. One-way ANOVA with post hoc analysis. (**C**) Immunofluorescence and (**D**) IHC analyses of Sema7a protein in the livers of WT and *Sema7a*^R145W^ homozygous mice. Normal rabbit IgG was used as the negative control. Original magnification, ×200. (**E**) Relative expression levels of Sema7a and its receptors integrin β1 and plexin C1 in whole-cell lysates (left) and membrane fractions (right) extracted from primary mouse hepatocytes. (**F**) N-glycosylated-Sema7a protein (~130 kDa) was detected in primary mouse hepatocytes. Western blot revealed the deglycosylated Sema7a (black arrow). (**G**) Relative levels of Sema7a and integrin β1 proteins in whole-cell lysates (left) and membrane fractions (right) from human hepatoma HepG2 cells after transfection with the plasmid for SEMA7A_WT or SEMA7A_R148W. (**H**) Co-immunoprecipitation analysis of protein interactions among Sema7a, PKCα, and integrin β1 in liver tissues from 10-week-old male WT and *Sema7a*^R145W^ homozygous mice. (**I**) Phosphorylated PKC-α (T638) and PKC-α protein levels in whole-cell lysates (left panel) and membrane fractions (right panel) from primary mouse hepatocytes. T638 and PKC-α protein levels in (**J**) HepG2 cells that were transfected with SEMA7A_WT or SEMA7A_R148W plasmid and (**K**) primary mouse hepatocytes following integrin β1 silencing. (**L**) Co-immunoprecipitation analysis of protein interaction between Sema7a and PKC-α in primary mouse hepatocytes after integrin β1 silencing. Data are representative images or expressed as the mean ± SD of each group from 3 separate experiments. The difference among the groups was determined by 1-way ANOVA with Tukey’s post hoc tests or by Kruskal-Wallis test with Dunn’s post hoc test analysis, and the difference between the groups was analyzed by Student’s *t* test. *P < 0.05 versus WT mice (cells); ^#^*P* < 0.05 versus *Sema7a*^R145W^ heterozygous mice (cells).

Real-time qPCR and Western blotting analyses demonstrated that the *Sema7a*^R145W^ mutation did not result in changes in hepatic Sema7a mRNA and protein expression in mice (Figure 6, A and B). However, immunofluorescence and immunohistochemistry indicated that the *Sema7a*^R145W^ homozygous mutation markedly increased Sema7a protein on hepatocyte membranes in mouse livers (Figure 6, C and D). Western blotting analysis of cell surface membrane extracts of primary mouse hepatocytes revealed that the *Sema7a*^R145W^ homozygous mutation dramatically increased the membrane-associated Sema7a and its receptor integrin β1 proteins, but not plexin C1, another Sema7a receptor (Figure 6E). Interestingly, treatment of whole primary mouse hepatocyte lysates with peptide N glycosidase F to remove N-glycans from glycoproteins (15) resulted in a markedly deglycosylated Sema7a protein band (Figure 6F, arrow) with an obviously decreased molecular weight along with a decrease in the ~130 kDa Sema7a protein band (Figure 6F), indicating that the ~130 kDa Sema7a protein was N-glycosylated. A similar pattern of N-glycosylated Sema7a and integrin β1 proteins was observed in HepG2 cells after transfection with the *SEMA7A*_R148W construct (Figure 6G). However, there was no significant difference in total Sema7a, integrin β1, and plexin C1 protein expression in these mutant mouse hepatocytes and HepG2 cells (Figure 6, E and G). Interestingly, treatment of mouse liver tissues with anti-Sema7a effectively precipitated not only integrin β1, but also PKC-α, a downstream molecule of integrin β1 signaling (16), particularly when using the *Sema7a*^R145W^ homozygous mouse liver tissues (Figure 6H). Hence, *Sema7a*^R145W^ mutation markedly increased its interaction with integrin β1 and PKC-α in mouse livers (Figure 6H). Moreover, the *Sema7a*^R145W^ mutation also increased the relative levels of PKC-α phosphorylation in whole-cell lysates and cell surface membrane fractions of *Sema7a*^R145W^ homozygous mouse hepatocytes and transfected HepG2 cells, indicating that the *Sema7a*^R145W^ homozygous mutation enhanced PKC-α activation (16), though it did not change its total protein expression (Figure 6, I and J). Furthermore, integrin β1 silencing dramatically reduced the levels of PKC-α phosphorylation and the interaction between Sema7a and PKC-α in primary *Sema7a*^R145W^ homozygous mouse hepatocytes, indicating that the increased activation of PKC-α by the *Sema7a*^R145W^ mutation depended on its receptor integrin β1 (Figure 6, K and L). Together, our data revealed that the *Sema7a*^R148W^ (human) mutation in hepatocytes increased the cell surface membrane localization of Sema7a and its receptor integrin β1 proteins and activated PKC-α signaling. Subsequently, the enhanced PKC-α activation stimulated FA and TG synthesis and FA uptake and modulated their metabolism in hepatocytes, leading to excessive accumulation of intrahepatic lipids and promoting the progression of NAFLD.

### The V334I, R302K, R66Q, N559Y, and T2M mutations in SEMA7A can also induce the activation of the PKC-α signaling and the expression of key genes for FA and TG synthesis and FA uptake in hepatocytes.

**Figure 7:**
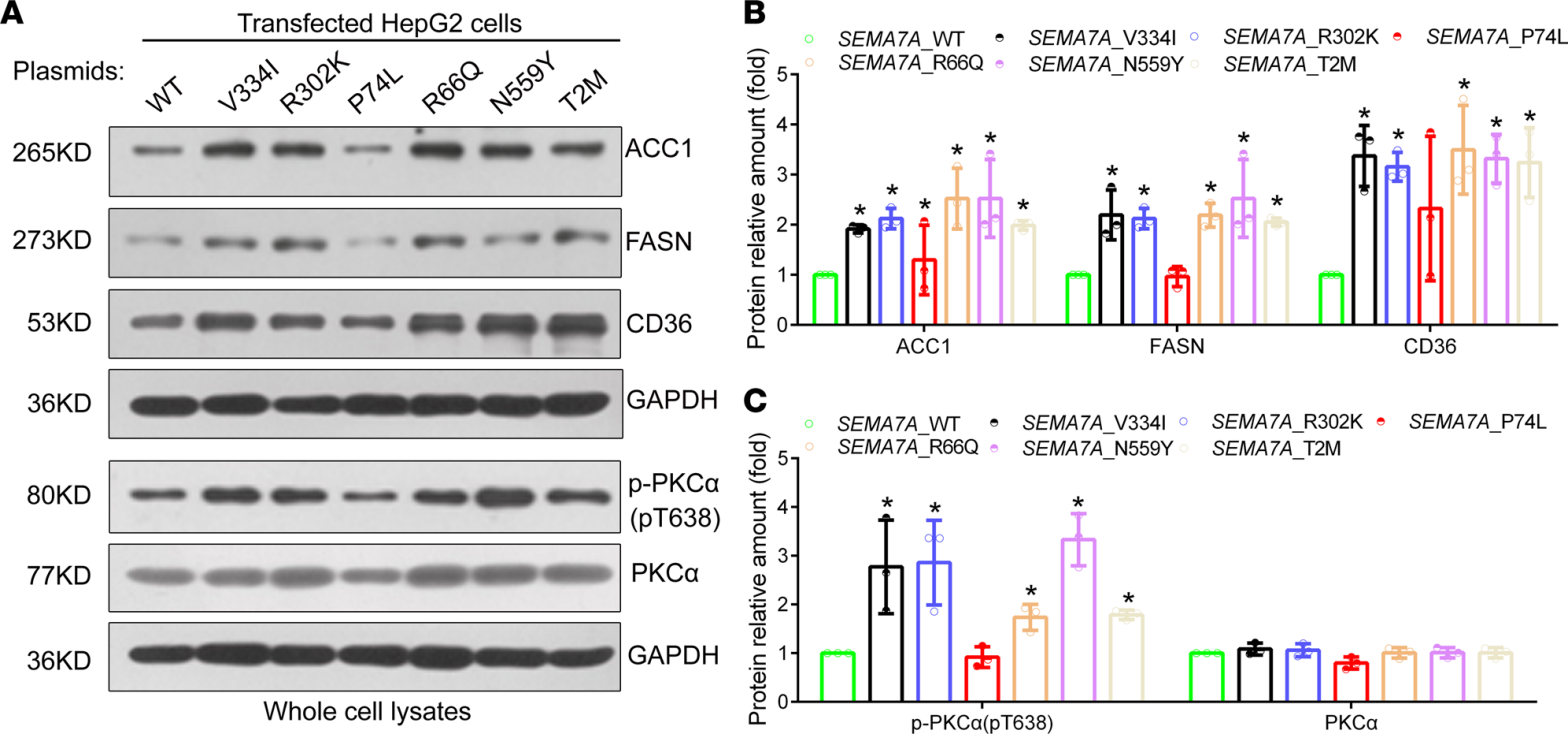
The *SEMA7A* mutation activates PKC-α signaling in hepatocytes. HepG2 cells were transfected with the plasmid for the expression of *SEMA7A*_WT or *SEMA7A*_V334I, _P302K, _P74L, _R66Q, _N559Y, or _T2M mutant, and the relative levels of ACC1, FASN, CD36, phosphorylated PKC-α (T638), and PKC-α expression in each group of cells were determined by Western blot. (**A**) Representative images of Western blot analyses. (**B**) Quantitative analysis of each mutant protein and (**C**) the relative levels of T638 and PKC-α in HepG2 cells from 3 separate experiments. The levels of each protein in the *SEMA7A*_WT–transfected cells were designated as 1. The data were analyzed by 1-way ANOVA with Tukey’s post hoc tests or by Kruskal-Wallis test with Dunn’s post hoc test analysis. *P < 0.05 versus the *SEMA7A*_WT cells.

To determine the functional significance of other mutations in SEMA7A, we generated serial plasmids for the expression of *SEMA7A*_V334I, *SEMA7A*_R302K, *SEMA7A*_P74L, *SEMA7A*_R66Q, *SEMA7A*_N559Y, and *SEMA7A*_T2M. Next, HepG2 cells were transfected with *SEMA7*_WT and each type of mutant plasmid (Supplemental Figure 5), and the effects on PKC-α phosphorylation and ACC1, CD36, and FASN expression were analyzed by Western blotting. As shown in Figure 7, A–C, the overexpression of *SEMA7A*_V334I, _R302K, _R66Q, _N559Y, and _T2M molecules, but not *SEMA7A*_P74L, significantly increased PKC-α phosphorylation and the expression of CD36, ACC1, and FASN in HepG2 cells. However, there was no single *SEMA7A* mutant that significantly altered the relative levels of PKC-α expression in HepG2 cells (Figure 7, A and C). Together, these data indicate that *SEMA7A*_V334I, _R302K, _R66Q, N559Y, and _T2M mutants may act similarly to the R148W mutant in regulating FA and TG synthesis and FA uptake in human hepatocytes.

## Discussion

**Figure 8:**
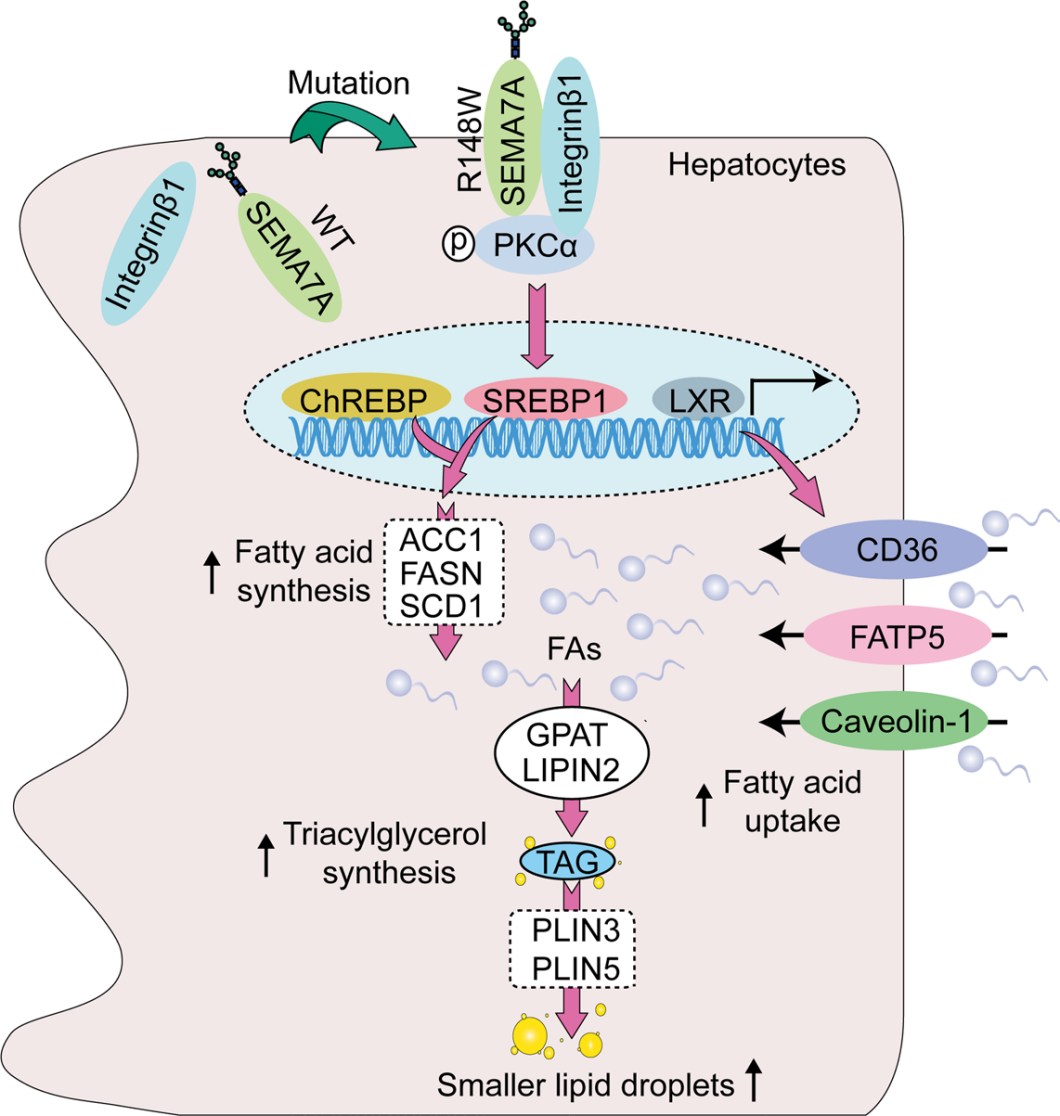
The potential mechanism by which the *SEMA7A*^R148W^ mutation causes lipid accumulation in hepatocytes. First, the mutation increases SEMA7A and its receptor integrin β1 proteins on the surface of cell membranes to promote PKC-α activation in hepatocytes. Second, the activated PKC-α signaling enhances the expression of transcriptional factors SREBP1 and ChREBP and nuclear receptor LXR, increasing FA and TG synthesis and FA uptake in hepatocytes. Finally, these increased the accumulation of small lipid droplets in the liver, leading to the development and progression of NAFLD.

This study reported a potentially novel genetic determinant of NAFLD, the R148W mutation in *SEMA7A*, and uncovered its pathophysiological mechanisms (Figure 8). Our study highlighted 4 potentially novel findings: (a) *SEMA7A* mutations were genetic risk factors for human NAFLD and its severity in humans (Tables 1 and 2); (b) the *Sema7a*^R145W^ mutation significantly increased steatosis severity and NAS in mice (Figure 1); (c) the *Sema7a*^R145W^ mutation markedly increased N-glycosylated Sema7a and its receptor integrin β1 proteins in hepatocyte surface membranes (Figure 6); and (d) the *Sema7a*^R145W^ mutation caused intrahepatic lipid accumulation by enhancing PKC-α signaling–stimulated FA and TG synthesis and FA uptake (Figures 2–6).

SEMA7A acts on integrin β1 and plexin C1 receptors to regulate multiple physiological and pathological processes (6–11). A recent study has shown that *Sema7a* deficiency reduces FA oxidation and oxidative phosphorylation in mouse peritoneal macrophages (12). Moreover, mutations in *SEMA7A* are associated with several disorders, such as Kallmann syndrome (10, 11). Here, we addressed whether mutations in S*EMA7A* affected lipid metabolism and contributed to the development of NAFLD. First, we identified 17 NAFLD patients with *SEMA7A* heterozygous mutation in a population of 470 patients with NAFLD, who had been evaluated by histological examination of biopsied liver specimens (Table 1). The *Sema7a*^R145W^ heterozygous mutation increased susceptibility to NAFLD and NAFLD severity in human patients and mouse models (Figure 1 and Table 2). Mechanistically, the *Sema7a*^R145W^ mutation markedly increased N-glycosylated SEMA7A and its receptor integrin β1 proteins on the cell membranes of hepatocytes, resulting in intrahepatic accumulation of small lipid droplets by enhancing PKC-α signaling–stimulated FA and TG synthesis and FA uptake (Figure 6). We also provided evidence that the *SEMA7A*^R148W^ mutation was a gain-of-function mutation, since (a) *Sema7a* deficiency reduces FA oxidation in mouse peritoneal macrophages and cholestatic liver injury (7), whereas the *Sema7a*^R145W^ homozygous mutation increased lipid accumulation and elevated levels of serum ALT and AST in mice (Figures 1 and 2); (b) the *Sema7a*^R145W^ mutation increased Sema7a and its receptor integrin β1 proteins on hepatocyte surface membranes; and (c) PKC-α, a downstream signaling molecule of integrin β1 (16), was detected in the immunoprecipitated Sema7a protein complex, and its phosphorylation was enhanced by the *Sema7a*^R145W^ mutation (Figure 6). In addition, KEGG analysis of the proteomics data between *Sema7a*^R145W^ homozygous mice and WT mice demonstrated that the oxidative phosphorylation pathway was the most abundant pathway (Figure 3H). Similarly, a recent study reported that the stability of oxidative phosphorylation subunits was reduced in a diet-induced mouse model of NAFLD (17), suggesting that the oxidative phosphorylation pathway may play a crucial role in NAFLD pathogenesis. Therefore, *Sema7a*^R145W^ mutation may also influence the oxidative phosphorylation pathway, leading to the progression of NAFLD. Nevertheless, this hypothesis will need to be addressed in the future.

Moreover, SEMA7A is also expressed in multiple tissues, including the brain, lung, intestine, kidney, bone, and immune system (5–14, 16, 18–24). Recent studies have shown that the upregulation of SEMA7A expression is significantly associated with multiple sclerosis (18), systemic sclerosis–related interstitial lung disease (19), rheumatoid arthritis (20), airway inflammation (21), colitis (22), systemic lupus erythematosus (23), and melanoma and other cancers (24). Thus, the *SEMA7A*^R148W^ mutation may also be the causative genetic factor for these diseases. Notably, SEMA7A is expressed in the cardiovascular system and is crucial for vascularization and angiogenesis (6). Atherosclerosis is characterized by the accumulation of lipids and extracellular matrix on the arterial wall (25). Our present data indicated that the *Sema7a*^R145W^ mutation caused lipid accumulation in the cell membranes of hepatocytes (Figure 2). Therefore, further studies are needed to ascertain whether the *SEMA7A*^R148W^ mutation contributes to arterial lipid metabolic dysfunction and atherosclerosis.

In conclusion, our potentially novel data indicated that the *SEMA7A*^R148W^ mutation was a novel genetic determinant of NAFLD and that PKC-α signaling–induced FA and TG synthesis and FA uptake were enhanced by the *SEMA7A*^R148W^ mutation. In our study, we discovered a molecular mechanism underlying the pathogenesis of NAFLD caused by the SEMA7A^R148W^ mutation. The inhibition of hepatic PKC-α signaling may lead to novel NAFLD therapies.

## Methods

### Patients with NAFLD.

Patients with NAFLD were enrolled from a well-characterized Prospective Epidemic Research Specifically of NASH cohort and diagnosed by histological examination of biopsied liver samples from December 2016 to July 2020 (13). Their body weight, height, waist circumference, and hip circumference were measured in light clothing by well-trained nurses in the morning. BMI (kg/m^2^) was calculated as body weight divided by the height squared. After an 8-hour overnight fast, patients’ blood samples were collected from the antecubital vein by experienced nurses. The levels of serum ALT, AST, GGT, ALP, TBIL, and DBIL in individual patients were analyzed using an automated analyzer (Abbott AxSYM) (13). The detailed methods for liver biopsies have been described in the Supplemental Methods. Genomic DNA was extracted from patients’ peripheral blood mononuclear cells, as described previously (13), and stored at –80°C.

### Single gene (SEMA7A) exon sequencing analysis in patients with NAFLD.

The DNA fragments for human *SEMA7A* exons (exon 1 to exon 14) were amplified by PCR using specific primers (Supplemental Table 1). The PCR products were subjected to Sanger sequencing analysis, which, together with data analysis, were supported technically by Beijing Genomics Institute. The data reported in this paper have been deposited in the OMIX, China National Center for Bioinformation/Beijing Institute of Genomics, Chinese Academy of Sciences (https://ngdc.cncb.ac.cn/omix: accession no. OMIX001290).

### OR estimation of SEMA7A heterozygous mutations for NAFLD.

The potential risk of *SEMA7A* heterozygous missense mutations for the development of NAFLD was estimated by OR and 95% CI through logistic regression analysis using Review Manager version 5.2. Data from East Asian patients (18,394 individuals) were extracted from the gnomAD and used as the controls. A 2-tailed *P* value of less than 0.05 was considered statistically significant.

### Baseline characteristic analysis of NAFLD patients with SEMA7A heterozygous mutations and their pair-matched controls.

We identified 17 NAFLD patients with *SEMA7A* heterozygous mutations in the 470-patient NAFLD cohort. However, 4 NAFLD patients with *SEMA7A* heterozygous mutations were excluded from further analyses because of the lack of pair-matched controls. Data from pair-matched controls with WT *SEMA7A* were extracted from the database for the study. The patients were pair-matched with the controls for age, sex, BMI, and the variation in *PNPLA3* (rs738409 C>G encoding PNPLA3 I148M), a risk factor for NAFLD (1, 2). Continuous data are expressed as mean ± SD and median (IQR), whereas categorical data are expressed as frequencies and percentages. The difference between groups was analyzed by χ^2^ test, paired 2-sample *t* test, and Wilcoxon’s signed-rank test where applicable.

### Generation and characterization of Sema7a^R145W^mutant mice and their sample collection.

*Sema7a*^R145W^ (c.433C>T) mutant C57BL/6J mice were designed and generated by Shanghai Model Organisms Center using the Cas9-targeted single guide RNA of 5′ ATGCCCGGAAGCCCAGCTGCTGG 3′ and a similar protocol described previously (26). The obtained F0 mice were characterized by PCR and sequencing using primer pairs: F1: 5′ GGAGGGAACATGAGTTTGCT 3′; R1: 5′ CCACATGACCACCGGCTACT 3′. Serum and liver samples were collected from 10-week-old WT (*n* = 10, 6 male/4 female), *Sema7a*^R145W^ heterozygous (*n* = 15, 9 male/6 female), and homozygous mice (*n* = 15, 9 male/6 female) as described previously (26).

### Sema7a^R145W^ heterozygous mice with HFD feeding.

Male 8-week-old *Sema7a*^R145W^ heterozygous and their age-matched WT C57BL/6J mice were randomized and fed with HFD (catalog D12492, Research Diets; WT mice, *n* = 5; heterozygous mice, *n* = 6) or NCD (catalog D12450J, Research Diets; WT mice, *n* = 5; heterozygous mice, *n* = 6) for 26 weeks. Their body weights were measured weekly. At the end of the HFD feeding, their blood samples were collected for preparing serum samples and the mice were euthanized. Their livers were dissected, imaged (Olympus Corporation), and weighed sequentially. The left lobes of the liver were frozen in liquid nitrogen, and the right lobes of the liver were fixed in formalin for subsequent paraffin-embedding and histological staining.

### GC/MS analysis of FA in mouse liver extracts.

Mouse liver samples were prepared from WT, *Sema7a*^R145W^ heterozygous, and *Sema7a*^R145W^ homozygous mice and subjected to GC/MS analysis of FA, as described previously (27, 28). The detailed procedures are described in the Supplemental Methods.

### Lipidomic analysis.

Hepatic lipids were extracted from WT, *Sema7a*^R145W^ heterozygous, and *Sema7a*^R145W^ homozygous mice (*n* = 4 per group) using the methyl *tert*-butyl ether (MTBE) method as described previously (29). Briefly, individual samples (30 mg each) were homogenized in 200 μL water and mixed sequentially with 20 μL internal lipid standard mixture, 800 μL of MTBE, and 240 μL of precooled methanol, followed by ultrasonication. The detailed procedures are described in the Supplemental Methods.

### Proteomic and bioinformatic analyses.

Liver samples from WT, *Sema7a*^R145W^ heterozygous, and *Sema7a*^R145W^ homozygous mice (*n* = 5 per group) were homogenized in a buffer (4% SDS, 100 mM Tris-HCl, 1 mM DTT, pH 7.6). The obtained proteins (200 μg, each) were digested with 4 μg trypsin (Promega) in 40 μL of 25 mM NH_4_HCO_3_ buffer overnight at 37°C, and the obtained peptides were desalted on C18 cartridges (Empore SPE, MilliporeSigma). The detailed procedures are described in the Supplemental Methods.

### Preparation and collection of primary mouse hepatocytes.

Primary mouse hepatocytes were isolated from 10- and 12-week-old WT and *Sema7a*^R145W^ mice using collagenase (Worthington Biochemical Corporation) perfusion as previously described (26, 30). The isolated hepatocytes were cultured in 5% FBS-Williams’ Medium E (Gibco, Thermo Fisher Scientific, catalog 12551) overnight. The cells were harvested and lysed, and their surface membranes were extracted and biotinylated for Western blot analysis. Similarly, their nuclei were extracted for TaqMan qPCR. The details of primer sequences and antibody information are listed in Supplemental Tables 7 and 8.

### Cell surface protein biotinylation and extraction.

Cell surface proteins from primary mouse hepatocytes and human HepG2 hepatoma cells were biotinylated using the EZ-Link Sulfo-NHS-SS-Biotin reagent (Thermo Fisher Scientific; catalog 21331), according to the supplier’s protocol (31–33). For biotinylation, cells (1 × 10^6^/well) were cultured in 6-well plates and washed 3 times with chilled phosphate-buffered saline (PBS). The cells were treated with 1.0 mg/mL Sulfo-NHS-SS-Biotin (Thermo Fisher Scientific) in chilled PBS for 60 minutes at 4°C to biotinylate the membrane proteins of the cultured cells. The cells were treated with 100 mM glycine in PBS for 10 minutes to terminate the biotinylation reactions and washed 3 times with ice-cold PBS (pH 8.0) to remove nonreacted biotinylation reagent. Subsequently, the cells were harvested and lysed, followed by centrifugation at 16,000*g* for 20 minutes at 4°C. The resulting supernatants (250 μg total proteins) were reacted with 200 μL of 10% streptavidin agarose (Thermo Fisher Scientific, 20347) and centrifuged at 500*g* for 5 minutes at 4°C, followed by washing 5 times with lysis buffer. The biotinylated proteins were eluted in 2× SDS sample buffer supplemented with DTT (0.02 g/mL) and analyzed by SDS-PAGE.

Other information on methods and materials is available in the Supplemental Methods.

### Study approval.

The study protocol for patients with NAFLD was approved by the Ethics Committee of the First Affiliated Hospital of Wenzhou Medical University. Written informed consent was obtained from each patient. For single-cell RNA sequencing, the study was carried out in accordance with the Declaration of Helsinki of the World Medical Association. The study protocol was reviewed and approved by the Institutional Ethics Review Board at the Southwest Hospital.

## Author contributions

Experiments were conceived and designed by JC. Experiments were performed by NZ, XZ, JD, QP, GL, JQ, ML, LL, and YC. Data analysis was done by JC, MHZ, QP, YP, XZ, JD, QX, and WYL. Special reagents/materials/analysis tools were provided by MHZ, WYL, QL, QW, and LZ. The manuscript was written by JC, SYC, and JLB and critically revised by XO.

## Supplementary Material

Supplemental data

## Figures and Tables

**Table 1:**
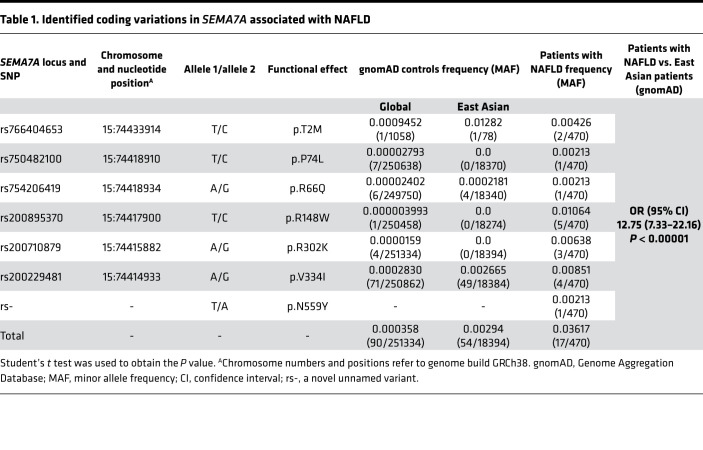
Identified coding variations in *SEMA7A* associated with NAFLD

**Table 2:**
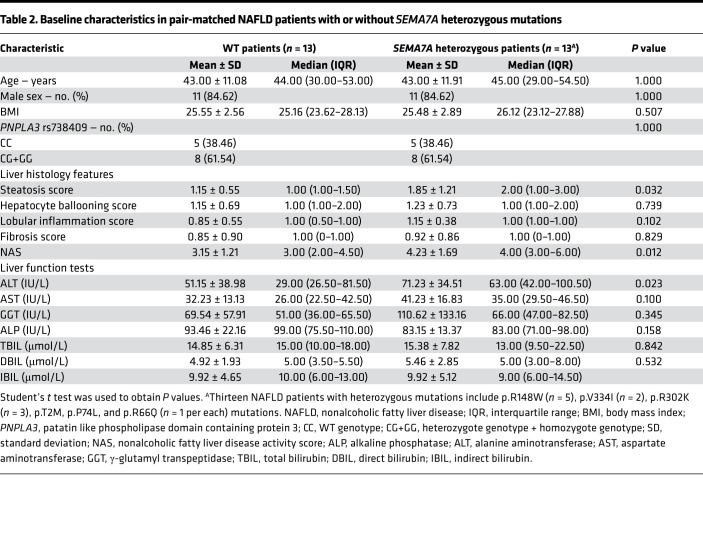
Baseline characteristics in pair-matched NAFLD patients with or without *SEMA7A* heterozygous mutations

## Version 1 08/08/2022

Electronic publication
