# Supplementary material for: SEMA7AR148W mutation promotes lipid accumulation and NAFLD progression via increased localization on the hepatocyte surface
Source: JCI Insight. 2022 Aug 8;7(15):e154113. doi: 10.1172/jci.insight.154113 (PMC9462498; doi:10.1172/jci.insight.154113)
Supplement: Supplemental data [file jciinsight-7-154113-s032.pdf]

Supplementary Documents

SEMA7A^{R148W} mutation promotes lipid accumulation and NAFLD progression by increasing its localization on hepatocyte surface

Nan Zhao[1]*, Xiaoxun Zhang[1]*, Jingjing Ding[1]*, Qiong Pan[1]*, Ming-Hua Zheng[2]*, , Wen-Yue Liu[3], Gang Luo[1], Jiaquan Qu[1], Mingqiao Li[1], Ling Li[1], Ying Cheng[1], Ying Peng[1], Qiaoling Xie[1], Qinglin Wei[1], Qiao Li[1], Lingyun Zou[4], Xinshou Ouyang[5], Shi-Ying Cai[6], James L. Boyer[6], and Jin Chai[1#]

[1]Department of Gastroenterology, Center for Metabolic Associated Fatty Liver Disease and Center for Cholestatic Liver Diseases, the First Affiliated Hospital (Southwest Hospital) of Third Military Medical University (Army Medical University), Chongqing, China

[2]NAFLD Research Center, Department of Hepatology, the First Affiliated Hospital of Wenzhou Medical University, Wenzhou 325000, China

[3]Department of Endocrinology, the First Affiliated Hospital of Wenzhou Medical University, Wenzhou 325000, China

[4]Bioinformatics Center, Department of Microbiology, Third Military Medical University, Chongqing 400038, China; Bao'an Maternal and Child Health Hospital, Jinan University, Shenzhen 518133, China

[5]Department of Internal Medicine, Section of Digestive Diseases, Yale University School of Medicine, New Haven, CT 06520, USA

[6]Department of Internal Medicine and Liver Center, Yale University School of Medicine, 333 Cedar Street, New Haven, CT 06520, USA

*These authors contributed equally to this study.

Footnote Page

Contact Information:

Jin Chai, M.D., Ph.D., Professor of Gastroenterology and Hepatology, Chongqing University School of Medicine and Third Military Medical University (Army Medical University). Address: Department of Gastroenterology, Center for Cholestatic Liver Diseases and Center for Metabolic Associated Fatty Liver Diseases, the First Affiliated Hospital (Southwest Hospital) of Third Military Medical University (Army Medical University) Chongqing, 400038, China; Tel: 86-23-68765331; Fax: 86-23-65410853; E-mail: jin.chai@cldcsw.org.

Funding:

This study was supported by grants from the National Natural Science Foundation of China (81922012, 81770583, 32171123), the Outstanding Youth Science Foundation of Chongqing (cstc2021jcyj-jqX0005), the Project of Chongqing Universities Innovation Research Group (2021cqspt01), and Natural Science Foundation of Southwest Hospital (Grant Nos. 2017YQRC-01 and XZ-2019-505-001).

Abbreviations:

Semaphorin 7A, SEMA7A; alanine transaminase, ALT; aspartate transaminase, AST; Peripheral blood mononuclear cells, PBMCs; non-alcoholic fatty liver disease NAFLD.

Competing interests:

The authors sate no conflicts of interest.

Contributors:

Experiments were conceived and designed by Jin Chai. Experiments were performed by Nan Zhao, Xiaoxun Zhang, Jingjing Ding, Qiong Pan, Gang Luo, Jiaquan Qu, Mingqiao Li, Ling Li, and Ying Cheng. Data analysis was done by Jin Chai, Ming-Hua Zheng, Qiong Pan, Ying Peng, Xiaoxun Zhang, Jingjing Ding, Qiaoling Xie, and Wen-Yue Liu. Special Reagents/Materials/Analysis tools were provided by Ming-Hua Zheng, Wen-Yue Liu, Qiao Li, Qinglin Wei and Lingyun Zou. Manuscript was written by Jin Chai, Shi-Ying Cai, James L. Boyer and critically revised by Xinshou Ouyang.

Table of contents

Content	Page
**Supplementary Materials and Methods**	5
**Supplementary Figures and Figure legends**	17
**Suppl.Fig.1.** H&E staining of the biopsied liver sections from NAFLD patients with heterozygous *SEMA7A* p.R148W, p.V334I, p.R302K, p.P74L, and p.T2M mutations, and their matched-control subjects.	17
**Suppl.Fig.2.** Single-cell RNA sequencing (5'-scRNA-seq) in human livers.	18
**Suppl.Fig.3.** The *Sema7a*^{R145W} mutation causes intrahepatic accumulation of small lipid droplets in female mice at 10-week of ages.	19
**Suppl.Fig.4.** IF analysis of SCD1 in the liver sections from NAFLD patients with *SEMA7A* heterozygous p.R148W, p.V334I, p.R302K, p.P74L, and p.T2M mutations, and their matched-control subjects.	20
**Suppl.Fig.5.** The data of transfection efficiency.	21
**Supplementary Tables and Table notes**	22
**Table S1.** The sequences of PCR primers for the single gene (*SEMA7A*) exons.	22
**Table S2.** Serum biochemistry of *Sema7a*^{R145W} heterozygous mice after HFD feeding.	23
**Table S3.** Targeted analysis of fatty acids by GC-MS in *Sema7a*^{R145W} mutant mouse livers.	24
**Table S4.** Quantitative lipidomic analysis in *Sema7a*^{R145W} mutant mouse livers.	26
**Table S5.** Quantitative analysis of different subclasses of triglycerides (TG) in *Sema7a*^{R145W} mutant mouse livers.	28
**Table S6.** Proteomics analysis of differentially expressed proteins in the NAFLD pathway in *Sema7a*^{R145W} mutant mouse livers.	29
**Table S7.** The sequences of Real time qPCR probes (TaqMan) and primers.	31
**TableS8.** Antibodies used in Western blot, co-immunoprecipitation, immunofluorescence and immunohistochemistry.	33
**Supplementary Reference**	34

Supplementary Materials and Methods

NAFLD Patients

NAFLD patients were enrolled from a well-characterized Prospective Epidemic Research Specifically of NASH (PERSONS) cohort and diagnosed by histological examination of biopsied liver samples from December 2016 to July 2020 (13). Their body weight, height, waist circumference and hip circumference were measured with light clothing by well-trained nurses in the morning. Body mass index (BMI, kg/m^2) was calculated as body weight divided by the height square. After an 8-hour overnight fasting, their blood samples were collected from the antecubital vein by experienced nurses. The levels of serum alanine aminotransferase (ALT), aspartate aminotransferase (AST), gamma-glutamyl transferase (GGT), alkaline phosphatase (ALP), total bilirubin (TBIL), and direct bilirubin (DBIL) in individual patients were analyzed using an automated analyzer (Abbott AxSYM, Park, IL) (13). The detailed methods used for liver biopsies have been described elsewhere (Suppl.Ref.1). Patients with NAFLD were diagnosed, based on histological steatosis grade > 5% and with definite NASH, based on the presence of NAFLD activity score (NAS) ≥ 5, according to the NASH Clinical Research Network classification (Suppl.Ref.2). Significant fibrosis was defined as liver fibrosis ≥ F2 on histology, while advanced fibrosis was defined by liver fibrosis ≥ F3, according to the Brunt's histologic criteria (Suppl.Ref.3). Genomic DNA was extracted from their blood mononuclear cells, as described previously (13) and stored at -80 ℃.

The single-cell RNA sequencing (5'-scRNA-seq) of human livers

Human liver collection, dissociation and preparation

Four patients with liver diseases were recruited from the Institute of Hepatobiliary Surgery, Southwest Hospital. Corresponding written informed consent was obtained from all patients. Patients underwent a liver resection with curative intent. This study was carried out in accordance with the Declaration of Helsinki (2008) of the World Medical Association. The study protocol was reviewed and approved by the Institutional Ethics Review Board at the Southwest Hospital, Chongqing, China. The resected liver tissues were stored in the GEXSCOPETM Tissue Preservation Solution (Singleron Bio, Nanjing, China). The specimens were washed with Hanks Balanced Salt Solution (HBSS，Gibco, Cat. No.14025-076) for 3 times and cut into small pieces. The tissue samples were digested with 2 ml GEXSCOPE® Tissue Dissociation Solution (Singleron) at 37℃ for 15 min in 15-ml centrifuge tube（Falcon, Cat. No.352095）with continual agitation. The generating cells were filtered through 40-micron sterile strainers（Falcon, Cat. No.352340）and centrifuged at 300 g for 5 min. The pellets were re-suspended in 1 ml PBS (Hyclone, Cat. No.SA30256.01). To remove red blood cells, the cell suspension was mixed with 2 mL RBC lysis buffer (Singleron) and centrifuged at 500 g for 5 min. The liver cells were stained with trypan blue (Bio-RAD, Cat. No.#1450013), counted and adjusted at 1×10^5 cells/mL PBS.

Single cell RNA sequencing

Single-cell suspensions were converted to barcoded scRNA-seq libraries using the

Chromium Single Cell 5'Library, Gel Bead & Multiplex Kit (10X Genomics, Pleasanton, USA), according to the manufacturer's instructions. Briefly, the cells were partitioned into Gel Beads in Emulsion in the Chromium™ Controller instrument where cell lysis and barcoded reverse transcription of RNA occurred. Libraries were prepared using 10x Genomics Library Kits and sequenced on Illumina HiSeq X with 150 bp paired end reads.

scRNA-seq quantifications and statistical analysis

Raw reads were processed to generate gene expression profiles using Cell Ranger v.3.0.2. Reads from the 10X library were mapped to GRCh38 with ensemble version 92 gene annotation. Reads with the same cell barcode, unique molecular identifier (UMI) and gene were grouped together to calculate the number of UMIs per gene per cell. The UMI count tables of each cellular barcode were used for further analysis. The different types of cells were identified and clustered using Seurat program (http://satijalab.org/seurat/, R package, v.3.0.1). The UMI count tables were loaded into R using read table function and analyzed at a resolution of 0.6 for FindClusters function. The resulting differentially expressed genes (DEGs) between different samples or consecutive clusters were identified with the FindMarkers function.

Liver histology

Liver sections (4 μm) were routine-stained with hematoxylin and eosin (H&E), Sirius Red and *Oil-Red* O, as previously described (26).

Measurement of triglyceride and cholesterol concentrations in mouse livers

The concentrations of triglycerides (TG) and cholesterols (Tch) in individual mouse livers were quantified using the Triglyceride Content Assay Kit, Total Cholesterol Content Assay Kit, and Free Fatty Acid Content Assay Kit (Boxbio Science & Technology, Beijing, China), according to the manufacturer's protocols. Individual mouse liver tissues were homogenized in the solvents provided and after centrifugation, the concentrations of TG and Tch in individual samples were measured in triplicate.

GC-MS analysis of fatty acids (FA) in mouse liver extracts

Mouse liver samples were prepared from *wild-type (*WT), *Sema7a*^{R145W} heterozygous, and homozygous mice and subjected to GC-MS analysis of FA, as described previously (27, 28). For analysis of medium- and long-chain FA, mouse liver samples from WT, *Sema7a*^{R145W} heterozygous, and homozygous mice (n=4 per group) were homogenized in 1 ml of chloroform methanol (2:1 v/v), and subjected to ultrasonication for 30 min. After centrifugation, the supernatant was reacted with 2 ml of 1% sulfuric acid in methanol, and incubated in an 80 °C water bath for 30 min to achieve FA esterification. Subsequently, the solution was mixed with 1 ml n-hexane and 5 ml water, and vortexed. Each supernatant sample (500 µl) was mixed with an internal standard (25 µl of 500 ppm methyl salicylate), and subjected to GS-MS analysis using an Agilent Model 7890A/5975C GC-MS system. The levels of medium- and long-chain FA were quantified, based on a calibration curve of Supelco 37-component FAME (FA methyl ester) mix at 0.5-1000 mg/L (Sigma-Aldrich).

The samples were separated by an Agilent DB-WAX capillary GC column (30 m × 0.25 mm ID × 0.25 μm) with a temperature of 50 ℃ for 3 min, increasing to 220 ℃ at a rate of 10 ℃/min, and maintaining at 220 ℃ for another 20 min. The carrier gas was helium (1.0 mL/min). Quality-control samples were used for testing and evaluating the stability and repeatability of the system. The electron bombardment ionization source, SIM (Selected ion Monitor) scanning mode, and electron energy were 70 eV. The GC-MS analysis of FA was technically supported by Shanghai Applied Protein Technology.

Lipidomic analysis

Hepatic lipids were extracted from WT, *Sema7a*^{R145W} heterozygous, and homozygous mice (n=4 per group) using the MTBE method as described previously (29). Briefly, individual samples (30 mg each) were homogenized in 200 μl water and mixed sequentially with 20 μl internal lipid standard mixture, 800 μl of MTBE and 240 μl of pre-cooled methanol, followed by ultrasonication. After being centrifuged at 14,000 g for 15 min at 10 ℃, the upper organic layer was collected and dried in nitrogen circulation. The extracts were re-dissolved in 200 μl of 90% isopropanol/ acetonitrile for further analysis.

The hepatic lipids were separated on a CSH C18 column (1.7 μm, 2.1 mm × 100 mm, Waters) using a linear gradient of 30-100% buffer B over 23 min at 300 μl/min, followed by equilibrating at 5% solvent B for 10 min (buffer A = acetonitrile-water

[6:4, v/v] with 0.1% formic acid and 0.1 mM ammonium formate; Buffer B = acetonitrile-isopropanol [1:9, v/v] with 0.1% formic acid and 0.1 mM ammonium formate). Mass spectra were acquired by Q-Exactive Plus in positive and negative mode, respectively. ESI parameters were optimized and preset for all measurements as follows: Source temperature, 300 ℃; Capillary Temp, 350 ℃, the ion spray voltage was set at 3000 V，S-Lens RF Level was set at 50% and the scan range of the instruments was set at m/z 200-1800. Qualitative analysis of lipids was processed using LipidSearch to search against the database. The lipidomic analysis was technically supported by Shanghai Applied Protein Technology.

Proteomic and bioinformatic analysis

Protein extraction and digestion

Liver samples from WT, *Sema7a*^{R145W} heterozygous, and homozygous mice (n=5 per group) were homogenized in SDT buffer (4% SDS，100 mM Tris-HCl，1 mM DTT，pH7.6). The obtained proteins (200 μg, each) were digested with 4 μg trypsin (Promega) in 40 μl of 25 mM NH_4HCO_3 buffer overnight at 37 ℃, and the obtained peptides were desalted on C18 cartridges (Empore™ SPE, Sigma). The peptide contents were estimated by UV light spectrum at 280 nm using an extinction coefficient of 1.1 of 0.1% (g/l) solution.

Isobaric labeling of the peptides and fractionation

The mixed peptide samples (100 μg each) were labeled with TMT reagents, according to the manufacturer's instructions (Thermo Scientific). The labeled peptides were fractionated using the High pH Reversed-Phase Peptide Fractionation Kit (Thermo Scientific). The dried peptide mixture was reconstituted, acidified with 0.1% TFA solution and loaded to the equilibrated, high-pH, reversed-phase fractionation spin column. The peptides were bound to the hydrophobic resin under aqueous conditions and desalted by washing the column with water. A step gradient of increasing acetonitrile concentrations in a volatile high-pH eluting solution was applied to elute the bound peptides into 10 different fractions collected by centrifugation. The collected peptide fractions were desalted on C18 cartridges.

LC-MS/MS analysis

LC-MS/MS analysis was performed on a Q Exactive mass spectrometer (Thermo Scientific) that was coupled to Easy nLC (Proxeon Biosystems, now Thermo Fisher Scientific) as described previously (Suppl.Ref.4). The peptides were loaded onto a reverse phase trap column (Thermo Scientific Acclaim PepMap100, 100 μm X2 cm, nanoViper C18) connected to the C18-reversed phase analytical column (Thermo Scientific Easy Column, 10 cm long, 75 μm inner diameter, 3 μm resin) in buffer A (0.1% Formic acid) and separated by a linear gradient of buffer B (84% acetonitrile and 0.1% Formic acid) at a flow rate of 300 nl/min controlled by IntelliFlow technology. The mass spectrometer was operated in positive ion mode. MS data were

acquired using a data-dependent top10 method dynamically choosing the most abundant precursor ions from the survey scan (300–1800 m/z) for HCD fragmentation. Automatic gain control (AGC) target was set to 3e6, and maximum inject time to 10 ms. Dynamic exclusion duration was 40.0 s. Survey scans were acquired at a resolution of 70,000 at m/z 200 and resolution for HCD spectra was set to 17,500 at m/z 200, and isolation width was 2 m/z. Normalized collision energy was 30 eV and the underfill ratio, which specified the minimum percentage of the target value likely to be reached at maximum fill time, and was defined as 0.1%. The instrument was run with peptide recognition mode enabled.

Database searching and statistical and bioinformatic analyses

The MS raw data from each sample were searched using the MASCOT engine (Matrix Science, London, UK; version 2.2) embedded into Proteome Discoverer 1.4 software for identification and quantitation analysis. Peak lists were searched against the mouse Swissprot_mouse_17042 using the following parameters: enzyme, trypsin; maximum missed cleavage, 2; fixed modification, carbamidomethylating (C); variable modification, oxidation (M) and TMT (protein N-terminus and K); mass tolerance at 20 ppm; MS/MS mass tolerance at 0.1 Da; false discovery rate (FDR) < 0.01. Significance was assessed by ratios of TMT reporter ion intensities in the MS/MS spectra. Peptides with changes greater than 1.2 fold ($p<0.05$) between samples were chosen for further bioinformatic analysis. The proteomic and bioinformatic analyses were technically supported by Shanghai Applied Protein Technology.

Plasmid construction and transfection in hepatoma HepG2 cells

Human hepatoma HepG2 cells (ATCC, Manassas, VA) were kindly provided by Prof. Cheng Qian (Southwest Cancer Center, Southwest Hospital, Third Military Medical University, Chongqing, China) and identified by STR. The plasmids of pcDNA3.1-*SEMA7A*_WT and *SEMA7A*_R148W for the expression of WT and non-fusion mutant proteins, respectively, were generated by Hunan Fenghui Biotechnology (Changsha, China). The pHACE-*PKCα*_WT and pHACE-*PKCα*_DN (dominant negative mutant) plasmids were obtained from Addgene (Cambridge, MA) (Suppl.Ref.5). HepG2 cells (10^5 cells/well) were cultured in 6-well plates overnight and transfected with individual types of plasmids using Fugene HD (Promega, USA), according to the manufacturer's protocol (Suppl.Ref.5). Similarly, mouse primary hepatocytes (QIAGEN, Cat. No. 301425) were transfected with the similar plasmids or integrin β1-specific siRNA or control scramble siRNA using the HiPerFect Transfection reagent (QIAGEN, Cat. No. 301705), according to the manufacturer's instruction. The sequences of integrin β1-specific siRNA were F: 5'-CUGUAAGUG CAAUUGUCAATT-3'; R: 5'-UUGACAAUUGCACUUACAGTT-3'.

RNA extraction, reverse transcription, and quantitative real-time polymerase chain reaction (RT-qPCR)

Total RNA was extracted from mouse liver tissues, primary mouse hepatocytes and human HepG2 cells using Trizol reagent (Invitrogen, Carlsbad, CA, USA) according to the manufacturer's instructions. The RNA samples were reverse-transcribed into

cDNA that was used as templates for RT-qPCR as described previously (Suppl.Ref4) using the TaqMan probes (Life Technologies, Carlsbad, CA, USA), SYBR kits and primers (Table.S7). *Glyceraldehyde-3-phosphate dehydrogenase* (*GAPDH*) was used as an internal control. The data were analyzed by $2^{-\Delta\Delta Ct}$.

Western blot analysis

Total liver tissue homogenates and cell lysates were prepared, as previously described (26). The biotinylation and isolation of cell surface membrane proteins and extraction of nuclear proteins were performed using the commercial kits (Thermo Scientific, Waltham, MA, USA), according to the manufacturer's instructions (26). Protein samples were separated by SDS-PAGE and transferred to PVDF membranes (0.22 µm). After being blocked, the membranes were incubated with primary antibodies (Table.S8). Interestingly, Sema7a proteins at ~130KD, ~100KD and ~70KD were clearly detected in mouse liver tissues (Fig.6B). However, a ~130KD N-glycosylated-Sema7a protein was mainly detected in whole cell lysates from primary mouse hepatocytes (Fig.6E&F).

Co-immunoprecipitation assay

The mouse liver tissues were homogenized in radio-immunoprecipitation assay (RIPA) buffer (Sigma-Aldrich) containing complete EDTA-free protease and phosSTOP phosphatase inhibitors (Roche, Palo Alto, CA, USA). The homogenates were subjected to co-immunoprecipitation (Co-IP) using anti-Sema7a antibody (Table.S8),

as described previously (Suppl.Ref5).

Peptide N glycosidase F (PNGaseF) treatment

The whole lysates of primary mouse hepatocytes (containing protease and phosphatase inhibitors) were denatured at 100 ℃ for 10 min using glycoprotein denaturing buffer, according to the manufacturer's instructions (P0704S; New England Biolabs, Ipswich, MA, USA). The denatured cell lysates were treated with, or without, PNGaseF (P0704S; New England Biolabs) at 1000U PNGaseF/20 μg total proteins at 37 ℃ for 1 h, and used for Western-blotting analysis.

Immunofluorescence and Immunohistochemistry analysis

Immunofluorescence (IF) and Immunohistochemistry (IHC) were performed as previously described (26) using the primary antibodies (Table.S8).

Statistical analysis

All experiment data are expressed as mean ± SD (or SEM) and were analyzed using Student's T test with two tailed and one-way ANOVA with Tukey's post hoc tests or by Kruskal-Wallis test with Dunn's post hoc tests analysis, using SPSS software (PASW Statistics 18, IBM; SPSS, Chicago, IL, USA). A P-value of <0.05 was considered statistically significant.

Study approval

The human samples study protocol were approved by the Ethics Committee of the First Affiliated Hospital of Wenzhou Medical University, Wenzhou, China (2016246) and Ethics Review Board at the Southwest Hospital, Chongqing, China (KY2021196). Written informed consent was obtained from each subject. All animal procedures and experiments were approved by the Animal Ethics Committee of Army Medical University, Chongqing, China (AMUWEC20201589).

Supplementary Figures.1-6 and Figure legends

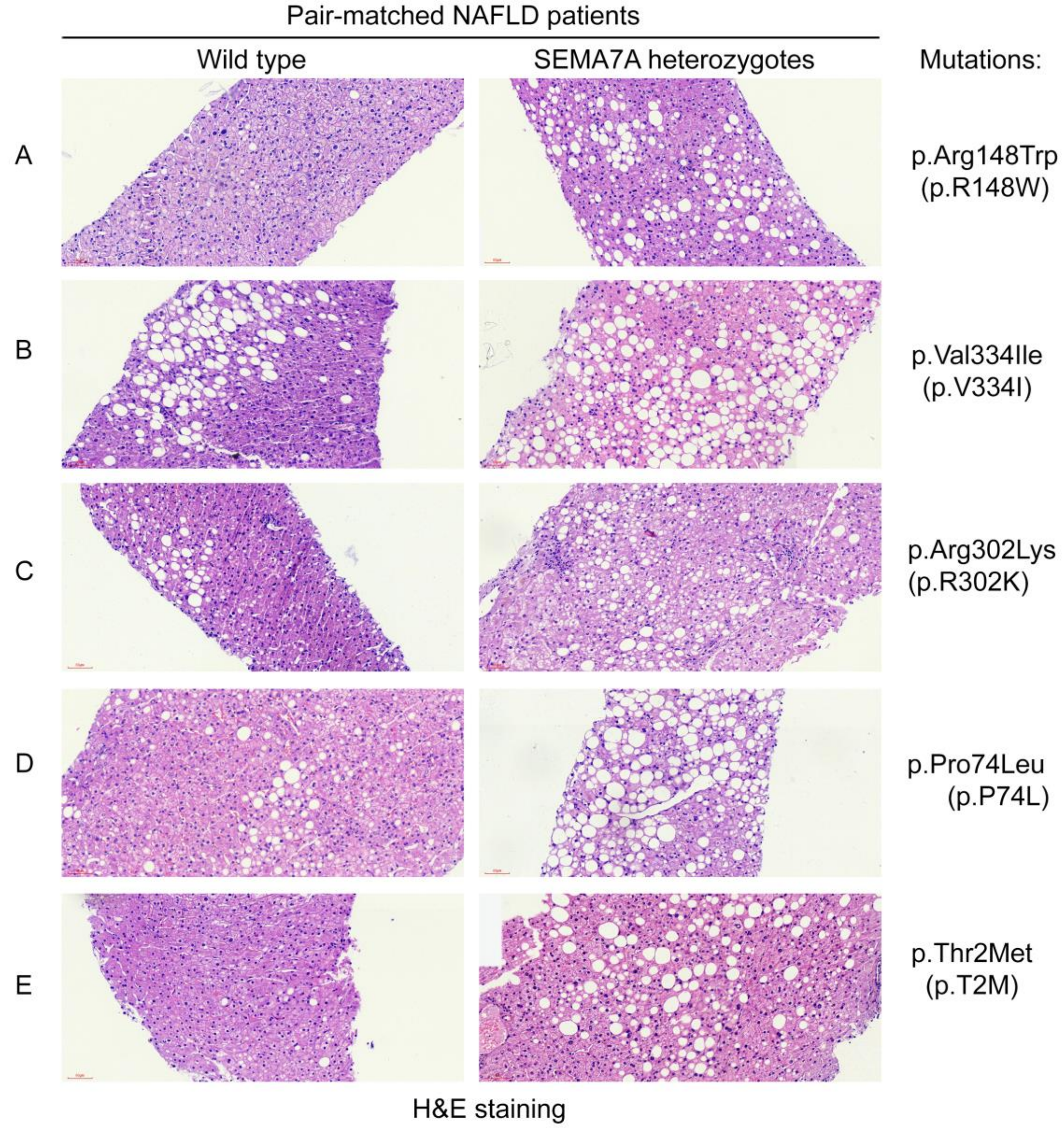

Suppl. Figure 1. H&E staining analysis of liver sections from NAFLD patients with a heterozygous *SEMA7A* p.R148W (A), p.V334I (B), p.R302K (C), p.P74L (D), or p.T2M (E) mutation, and their matched-control patients.

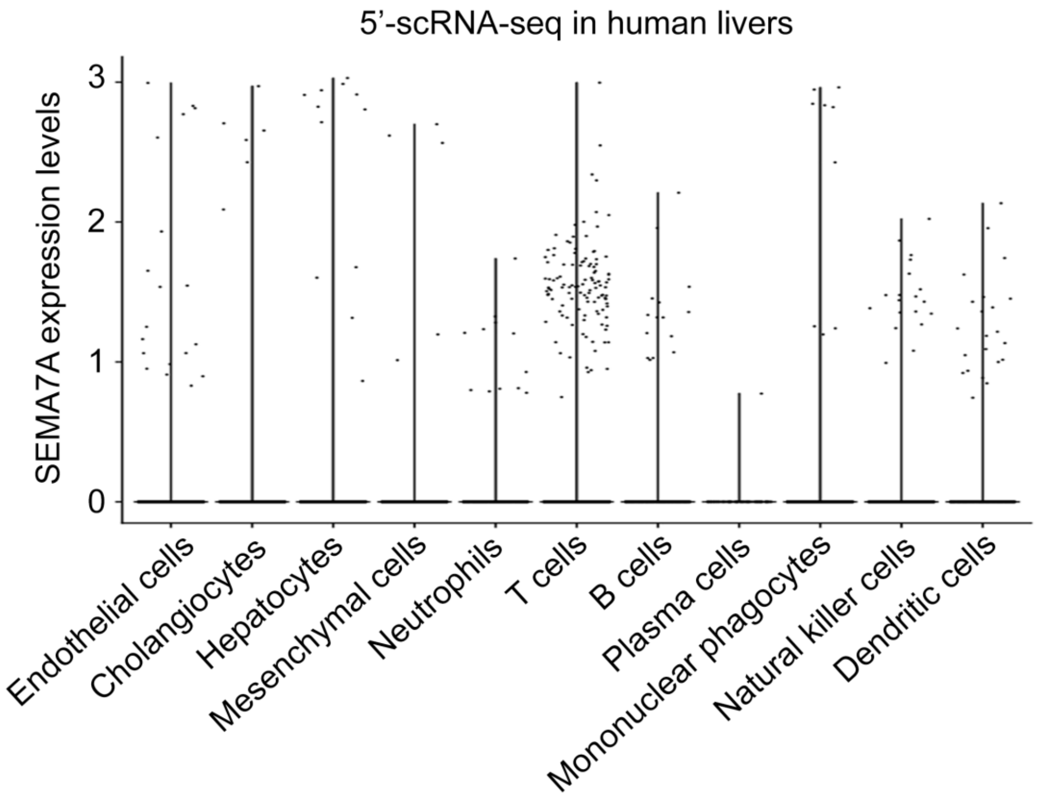

Suppl. Figure 2. Single-cell RNA sequencing (5'-scRNA-seq) of *SEMA7A* in human liver cells (n=4). SEMA7A was expressed in hepatocytes, cholangiocytes, endothelial cells, mesenchymal cells, neutrophils, T cells, B cells, plasma cells, mononuclear phagocytes, natural killer cells, and dendritic cells.

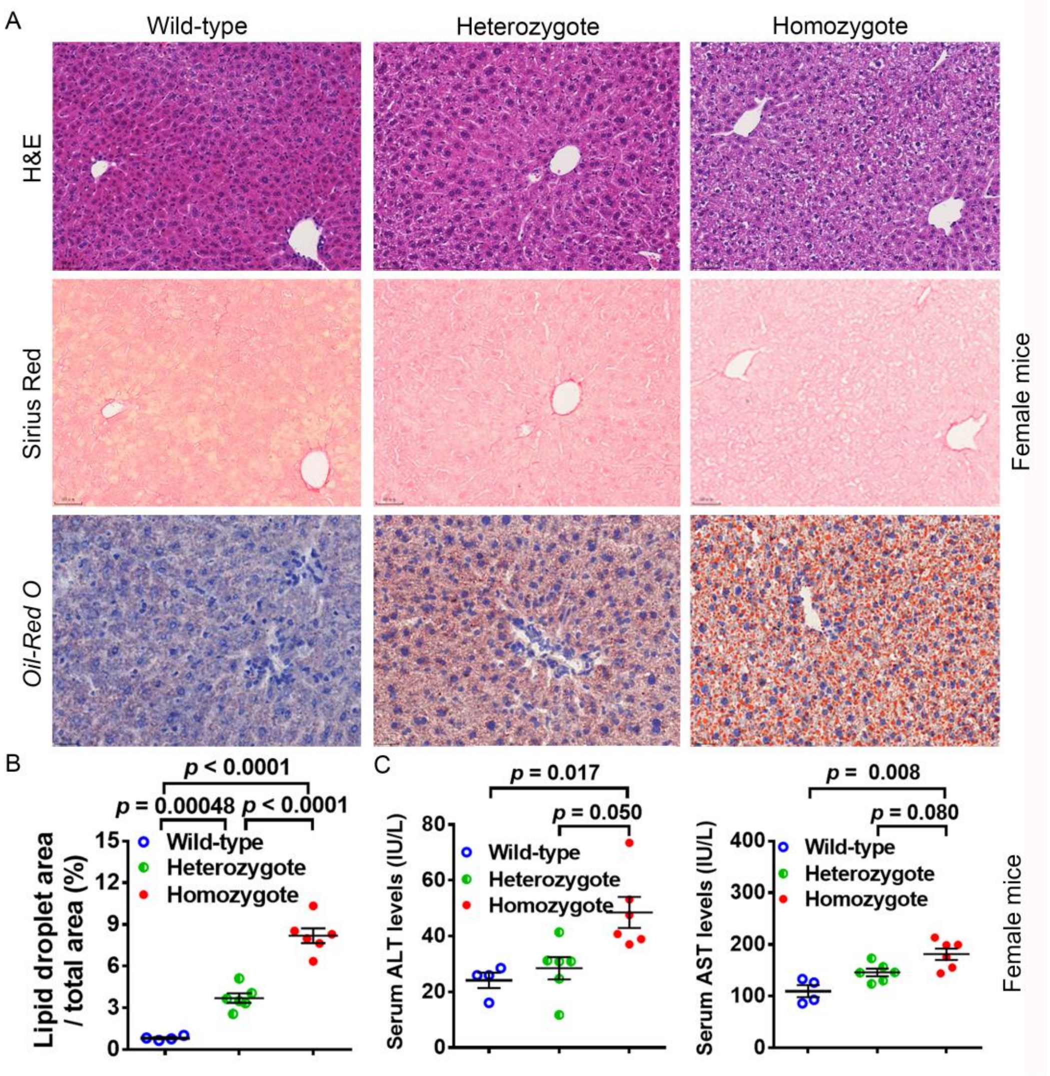

Suppl. Figure 3. The *Sema7a*^{R145W} mutation causes intrahepatic accumulation of small lipid droplets in female mice at 10-week of ages. (A) Representative images (magnification x 200) of H&E staining, *Sirius Red* staining, and *Oil-Red* O staining in WT, *Sema7a*^{R145W} heterozygous and homozygous female mice at the age of 10 weeks. (B) Analysis of lipid droplets in the *Oil-Red* O–stained liver sections of WT (n=4), *Sema7a*^{R145W} heterozygous (n=6) and homozygous female mice (n=6). (C) The levels of serum ALT and AST in *Sema7a*^{R145W} WT (n=4), heterozygous (n=6) and homozygous female mice (n=6). The data were analyzed by one-way ANOVA with Tukey's post hoc tests or by Kruskal-Wallis test with Dunn's post hoc tests analysis.

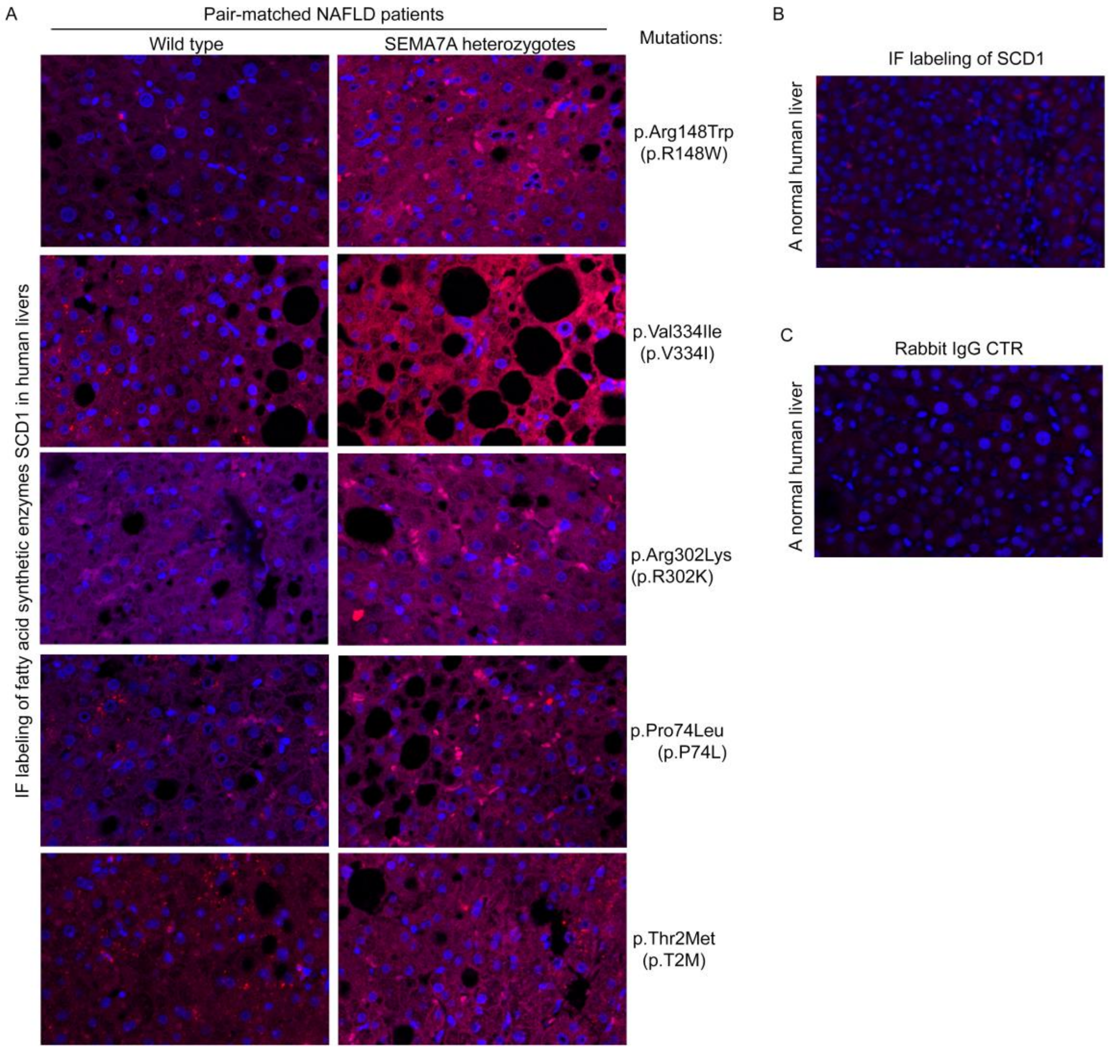

Suppl. Figure 4. IF analysis of the expression of SCD1, a fatty acid synthetic enzyme (A) in the liver sections from NAFLD patients with *SEMA7A*^{R148W} heterozygous mutations, p.V334I, p.R302K, p.P74L, and p.T2M mutations (right), and their matched-control patients (left). (B) IF analysis of SCD1 expression in the liver from a healthy individual. (C) Normal mouse liver section with rabbit IgG served as the negative control.

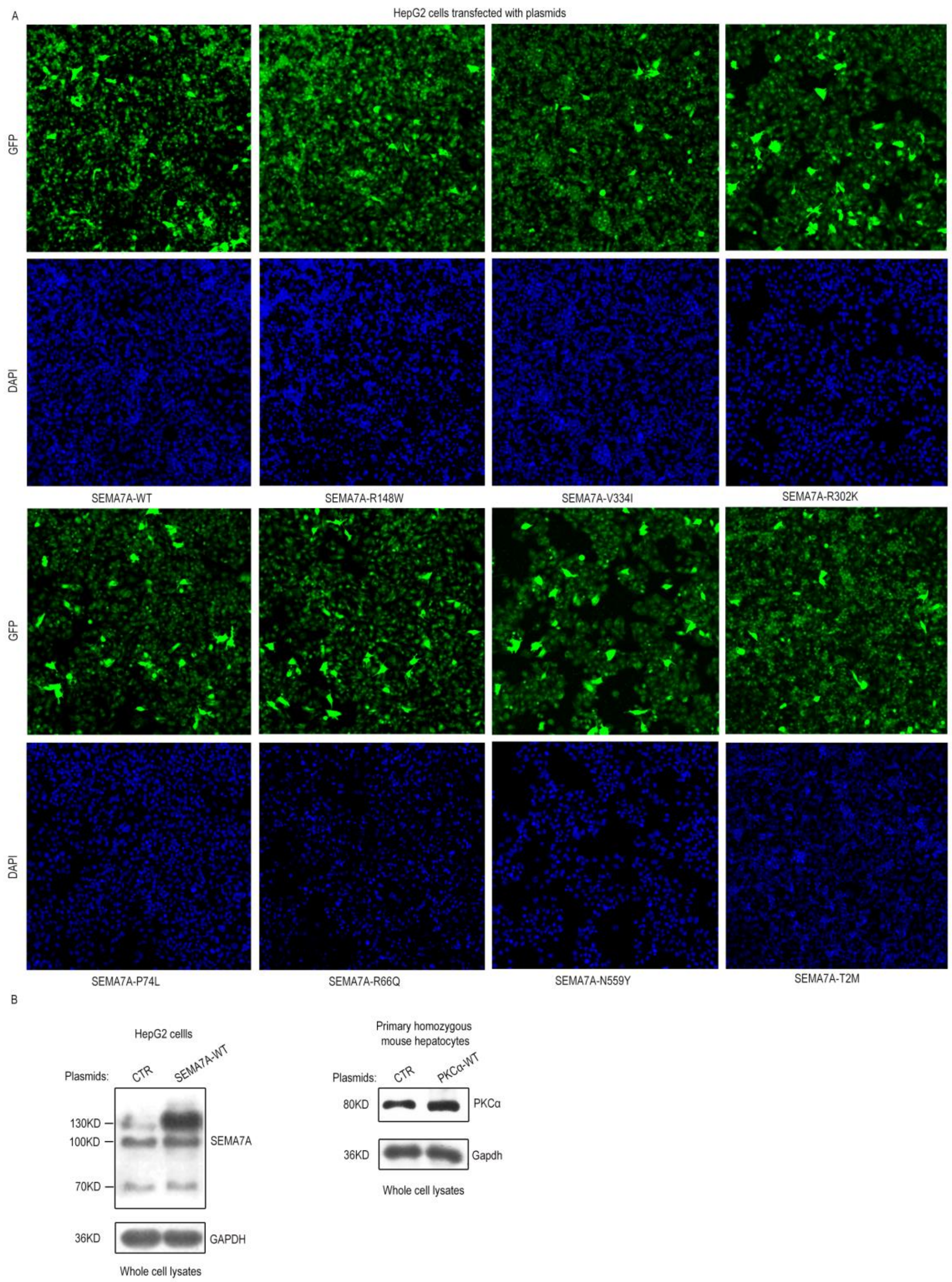

Suppl. Figure 5. The efficiency of transfection with GFP-SEMA7A_WT, R148W, V334I, R302L, P74L, R66Q, N559Y, or T2M construct in HepG2 cells and PKCα_WT construct in primary mouse hepatocytes were analyzed by fluorescent microscopy (A) and Western-blotting (B). Nuclei were stained with DAPI (blue).

Supplementary Tables.1-8

Table S1. The sequences of PCR primers for the *SEMA7A* exons

***Human SEMA7A* exons**	**Forward primer**	**Reverse primer**	**Products**
*SEMA7A*_Exon1	AAAGCCAGTGAGGCGAGAAAA	CGAGCCAGAGGACCCAGAGTG	670bp
*SEMA7A*_Exon2	CCCTGGTCCTCCTGCCTTAG	GGCCCATTAGCTCCTGTTTT	740bp
*SEMA7A*_Exons 3-4	GAGCCCATGCCTGGTCTTCT	CAGCACTGCCCTACCTTCAAAC	956bp
*SEMA7A*_ Exons 5-6	ATGCTAAGCCCTTCTGACACC	CCCAGCCAACCCTAACTTCT	907bp
*SEMA7A*_Exon7	GGAGCAGAGGAACAGACAAG	GATGAGACTCCAGCAAGCAG	1023bp
*SEMA7A*_Exon8	TGCTTGCTGGAGTCTCATCTGG	ATACATCGCACTGGCTGTCCC	543bp
*SEMA7A*_Exons 9-10	GGTTGGGTAGAGCAGATGGC	ATCTTCTTCCCTGAGTCAGACAT	946bp
*SEMA7A*_ Exons11-13	CTGCCCTTAGGCTCCCACAT	TTTCTCCCGTCTCGCTCATC	1061bp
*SEMA7A*_Exon14	GCCTTGTATCTGCTGATCCTCT	ACAGTCGGTGCCCTCATTCT	997bp

Table S2. Biochemistry analyses of serum samples from *Sema7a*^{R145W} heterozygous mice after feeding with HFD for 26 weeks

	Normal chow diet (NCD)		High-fat diet (HFD)	
	Wild type (n = 5)	Heterozygote (n = 6)	Wild type (n = 5)	Heterozygote (n = 6)
**Serum ALT (IU/L)**	15.28 ±3.60	54.60 ±20.64	348.64 ±40.28*,#	608.00 ±84.70*,#,$
**Serum AST (IU/L)**	82.48 ±13.94	152.20 ±49.73	389.60 ±32.35*,#	521.20 ±53.43*,#,$
**Liver tissue TG (mg/g)**	46.46 ±8.60	73.80 ±11.28	428.34 ±38.81*,#	588.17 ±47.01*,#,$
**Liver tissue Tch (mg/g)**	0.90 ±0.18	1.30 ±0.19*	1.83 ±0.24*,#	2.86 ±0.27*,#,$

Notes: Values are means ± SD. **P* < 0.05 versus NCD-WT mice, #*P* < 0.05 versus NCD-heterozygote mice, $*P* < 0.05 versus HFD-WT mice. The data were analyzed by the data were analyzed by one-way ANOVA with post-hoc analysis.

Abbreviations: ALT, alanine aminotransferase; AST, aspartate aminotransferase; TG, triglycerides; Tch, total cholesterol. Normal chow diet (NCD) was set as the control diet.

Table S3. GC-MS analysis of fatty acids in *Sema7a*^{R145W} mouse livers

	Wild type (n = 4)	Heterozygote (n = 4)	Homozygote (n = 4)
C8:0	0.020 ±0.003	0.034 ±0.040	0.034 ±0.011
C10:0	0.042 ±0.021	0.047 ±0.050	0.051 ±0.026
C11:0	0.010 ±0.002	0.019 ±0.023	0.013 ±0.005
C12:0	0.964 ±0.822	0.128 ±0.050	1.079 ±0.979
C13:0	0.114 ±0.075	0.060 ±0.041	0.144 ±0.053
C14:0	17.350 ±10.123	10.794 ±3.066	73.195 ±38.096*,#
C14:1N5	0.957 ±0.315	1.183 ±1.287	3.085 ±1.798
C15:0	8.421 ±2.177	12.207 ±1.929*	18.537 ±2.857*,#
C15:1N5	1.790 ±0.492	2.213 ±0.656	3.182 ±1.542
C16:0	1973.204 ±354.354	1945.687 ±633.287	3406.793 ±576.152*,#
C16:1N7	70.147 ±32.929	98.430 ±19.649	483.236 ±186.855*,#
C17:0	25.253 ±2.669	34.833 ±5.43*	43.621 ±5.678*
C17:1N7	5.517 ±1.178	14.338 ±3.794	32.495 ±8.015*,#
C18:0	1098.039 ±118.037	988.202 ±273.145	1703.160 ±354.196*,#
C18:1N9	941.401 ±338.284	848.219 ±171.781	3480.496 ±187.28*,#
C18:1TN9	2.342 ±1.739	3.951 ±2.988	2.150 ±1.662
C18:2N6	2024.044 ±542.839	1385.506 ±348.308	2819.672 ±504.352#
C18:2TTN6	0.745 ±0.271	1.190 ±0.246	2.410 ±0.618*,#
C18:3N3	49.362 ±29.158	20.565 ±7.614	74.466 ±22.679#
C18:3N6	30.485 ±14.471	20.689 ±10.026	48.505 ±13.908#
C20:0	15.874 ±6.361	24.221 ±5.495	17.094 ±18.604
C20:1N9	25.683 ±6.694	36.862 ±11.208	78.087 ±31.233*,#

C20:2N6	27.095±3.251	36.455±11.09	65.160±5.514*,#
C20:3N3	4.349±0.485	6.243±1.879	8.741±0.79*,#
C20:3N6	54.413±21.456	105.083±8.557*	193.481±22.741*,#
C20:4N6	936.929±95.268	990.954±238.468	1460.112±321.962*
C20:5N3	2.878±0.374	2.747±1.017	2.940±0.690
C21:0	0.621±0.098	1.443±0.346*	1.488±0.200*
C22:0	124.903±12.018	99.165±45.649	102.948±13.350
C22:1N9	6.918±0.829	12.871±10.744	11.911±5.333
C22:2N6	0.602±0.058	1.101±0.183	1.287±0.992
C22:4N6	18.584±4.689	30.643±11.526	54.232±10.947*,#
C22:5N3	87.597±28.395	90.674±65.641	102.885±19.338
C22:5N6	9.000±2.078	19.418±10.895	34.491±6.273*,#
C22:6N3	241.682±29.898	221.727±192.751	213.783±60.599
C23:0	0.281±0.045	0.475±0.124*	0.348±0.062
C24:0	2.163±0.482	3.633±2.135	3.554±0.571
C24:1N9	1226.144±165.916	773.356±291.842	1165.871±233.441
Total_SFA	3267.260±396.630	3120.950±967.990	5372.060±827.610*,#
Total_MUFA	2280.900±517.430	1791.420±476.100	5260.510±321.610*,#
Total_PUFA	3487.760±642.010	2933.000±898.430	5082.170±888.950#

Notes: Values are means ± SD (μg/g of liver). *$P < 0.05$ versus WT mice, #$P < 0.05$ versus heterozygous mutant mice. The data were analyzed by one-way ANOVA with Tukey's post hoc tests or by Kruskal-Wallis test with Dunn's post hoc tests analysis.

Table S4. Quantitative lipidomic analysis in *Sema7a*^{R145W} mouse livers

	**Wild type (n = 4)**	**Heterozygote (n = 4)**	**Homozygote (n = 4)**
AcCa	0.68±0.15	0.32±0.11*	0.37±0.06*
Cer	123.24±39.32	146.43±25.60	96.07±6.07
CerG1	5.70±1.18	5.71±1.71	6.68±0.85
CerG2	2.19±0.72	2.05±0.13	1.96±0.21
CL	101.20±37.03	76.01±33.88	67.66±13.16
Co	3.01±2.05	4.09±1.52	4.51±0.94
DG	25095.36±16751.92	6566.13±2152.46	16227.88±2275.63
FA	45.42±8.83	32.43±12.86	51.63±10.93
LPC	1395.63±149.39	1827.42±531.39	2426.38±1101.83
LPE	880.82±95.12	1097.6±382.00	1231.64±425.71
LPG	11.38±1.79	17.77±9.45	28.09±12.87
LPI	20.28±1.99	19.66±4.09	22.50±7.13
LPS	0.44±0.49	1.04±0.84	0.92±1.18
MGDG	42.80±10.74	51.34±16.28	44.92±5.70
MGMG	0.09±0.05	0.11±0.07	0.27±0.02*,#
PA	546.46±116.91	476.24±88.64	698.68±102.49#
PC	7655.57±1024.28	7617.38±365.94	8274.47±274.46
PE	4082.79±724.99	4315.21±684.40	3691.78±470.75
PG	619.73±272.69	1001.57±316.37	613.15±194.12
phSM	0.97±0.19	1.22±0.44	1.36±0.22
PI	457.08±33.81	457.07±68.42	501.58±98.97
PIP	18.32±5.35	13.90±5.19	30.49±9.38#

PS	26073.20±3211.83	24511.71±2837.04	29179.95±6578.25
SM	1881.08±238.03	1167.45±125.88*	1462.35±67.40*
So	1.03±0.85	3.04±1.47*	1.22±0.15#
SQDG	22.83±5.66	37.60±19.25	43.21±21.09
TG	5228.93±3437.15	2313.91±1538.44	12559.57±2489.40*,#
WE	0.27±0.18	0.41±0.19	0.20±0.03

Notes: Values are means ± SD (μg/g of liver). *$P < 0.05$ versus WT mice, #$P < 0.05$ versus heterozygous mutant mice. The data were analyzed by one-way ANOVA with Tukey's post hoc tests or by Kruskal-Wallis test with Dunn's post hoc tests analysis.

Table S5. Quantitative analysis of different subclasses of triglycerides (TG) in *Sema7a*^{R145W} mouse livers

	**Wild type (n = 4)**	**Heterozygote (n = 4)**	**Homozygote (n = 4)**
TG(16:0/18:1/22:4)	19.81±13.10	8.36±3.06	71.18±20.69*,#
TG(18:0/16:0/18:1)	75.29±52.63	68.65±72.14	220.14±87.08*,#
TG(16:0/16:0/16:1)	28.96±26.54	38.58±46.27	93.26±26.22
TG(18:1/18:1/18:1)	155.46±90.33	129.46±159.24	353.52±58.05#
TG(16:0/16:0/18:1)	131.30±75.69	124.01±105.48	295.19±63.65#
TG(16:0/18:2/20:3)	124.95±68.75	68.78±75.54	335.99±121.59*,#
TG(16:0/18:1/20:3)	125.13±79.70	100.73±90.27	369.08±107.19*,#
TG(16:0/18:1/22:4)	24.68±11.37	21.86±21.37	99.71±32.69*,#
TG(20:1/18:1/18:2)	15.45±14.44	8.49±4.69	47.89±12.43*,#
TG(16:0/18:1/18:1)	382.96±192.97	321.26±127.38	1801.58±463.60*,#
TG(16:0/17:1/18:1)	9.92±7.44	14.25±7.69	141.24±50.97*,#
TG(17:0/18:1/18:1)	13.72±10.51	15.28±10.77	102.86±36.65*,#
TG(16:0/18:1/20:1)	75.68±35.00	103.44±92.43	399.70±140.71*,#
TG(16:0/17:1/18:2)	17.82±17.73	6.11±3.42	81.04±29.55*,#
TG(16:0/18:1/20:4)	60.16±54.19	17.97±3.30	220.69±21.79*,#
TG(17:0/18:1/18:2)	21.09±20.27	9.16±4.33	79.16±29.38*,#
TG(16:0/16:0/18:3)	123.76±110.86	56.70±51.36	491.74±163.87*,#
TG(16:0/18:1/18:3)	473.03±268.00	127.54±82.42	1250.32±291.87*,#
TG(16:0/16:0/20:3)	777.22±362.52	376.86±110.00	2205.43±559.22*,#

Notes: Values are means ± SD (μg/g of liver). *P < 0.05 versus WT mice, #P < 0.05 versus heterozygous mutant mice. The data were analyzed by one-way ANOVA with Tukey's post hoc tests or by Kruskal-Wallis test with Dunn's post hoc tests analysis.

Table S6. Proteomics analysis of the differentially expressed proteins in the NAFLD pathway in *Sema7a*^{R145W} mouse livers

	Wild type	Heterozygote	Homozygote
Acc1	0.75±0.01	1.03±0.08*	1.22±0.07*,#
Plin3	0.85±0.02	1.09±0.01*	1.06±0.03*
Casp3	0.83±0.02	1.03±0.07*	1.14±0.06*
Fasn	0.70±0.01	1.01±0.04*	1.32±0.17*
Acc2	0.75±0.03	1.07±0.13*	1.21±0.13*
Scd1	0.44±0.08	1.18±0.04*	1.27±0.14*
Cd36	0.91±0.09	0.90±0.16	1.26±0.12*,#
Ndufv3	1.18±0.11	0.97±0.04*	0.91±0.06*
Cox5a	1.15±0.05	0.94±0.06*	0.92±0.03*
Cyp3a25	1.15±0.09	1.00±0.01*	0.88±0.04*
Ndufb2	1.12±0.09	1.00±0.05	0.90±0.02*
Ndufab1	1.14±0.04	1.02±0.36	0.81±0.05
Ndufa2	1.16±0.01	0.96±0.06*	0.88±0.08*
Mtco2	1.18±0.02	0.89±0.05*	0.94±0.07*
Ndufa3	1.12±0.01	0.99±0.07*	0.93±0.02*
Ndufb10	1.13±0.04	0.97±0.08*	0.91±0.04*
Ndufa13	1.16±0.04	0.93±0.03*	0.95±0.05*
Cyp3a11	1.55±0.19	0.71±0.09*	0.83±0.19*
Ndufa1	1.28±0.07	0.96±0.13*	0.78±0.04*

Ndufs3	1.11±0.04	0.98±0.04*	0.90±0.04*
Cyp2e1	1.20±0.02	0.91±0.07*	0.90±0.04*
Ndufb8	1.13±0.08	0.97±0.09	0.92±0.07*
Ndufb3	1.14±0.08	0.94±0.05*	0.93±0.03*
Cycs	1.09±0.10	1.00±0.13	0.88±0.02
Ndufa4	1.16±0.06	0.92±0.02*	0.94±0.02*
Cox7a2l	1.14±0.03	0.94±0.08*	0.95±0.02*
Uqcrq	1.15±0.08	0.96±0.12	0.93±0.03*
Uqcr10	1.12±0.06	0.97±0.08	0.90±0.03*
Uqcrh	1.12±0.05	0.98±0.13	0.90±0.03*
mt-Co3	1.07±0.08	1.04±0.06	0.89±0.06*

Notes: The data are the proteins associated with NAFLD; Other proteomics data can be found in raw data. N = 5 per group. *$P < 0.05$ versus WT mice, #$P < 0.05$ versus heterozygous mutant mice. The data were analyzed by one-way ANOVA with Tukey's post hoc tests or by Kruskal-Wallis test with Dunn's post hoc tests analysis.

Table S7. The sequences of real time qPCR probes (TaqMan) and primers.

Gene	Sequences (5’→3’)	Species/Source
Acaca	Forward: 5’- ccaggagacgcaagtccatc-3’	Mouse/Primers (SYBR)
	Reverse: 5’- ctctgtgttctcacgggctt-3’	NM_133360.2
Fasn	Proprietary to ABI	Mouse/Mm00662319_m1
Scd1	Forward: 5’- gagaagggcggaaaactgga-3’	Mouse/Primers (SYBR)
	Reverse: 5’-gagcaccagagtgtatcgca-3’	NM_009127.4
Gpat	Forward: 5’- gtctccagcttccagctaca-3’	Mouse/Primers (SYBR)
	Reverse: 5’-cagggctttgcttactggtc-3’	NM_001356285.1
Lipin1	Forward: 5’- aagagactgacaacgatcagga-3’	Mouse/Primers (SYBR)
	Reverse: 5’-ttccccagagaaccagtggat-3’	NM_172950.3
Lipin2	Forward: 5’- tgaagtggcggctctctattt-3’	Mouse/Primers (SYBR)
	Reverse: 5’-agagggttacatcaggcaagt-3’	NM_022882.4
Cd36	Proprietary to ABI	Mouse/Mm00432403_m1
Fatp2	Forward: 5’- tcgtggaggtctgaagtcact-3’	Mouse/Primers (SYBR)
	Reverse: 5’-gatggttgccgcttttggaa-3’	NM_011978.2
Fatp5	Proprietary to ABI	Mouse/Mm00447768_m1
Caveolin1	Forward: 5’- agcatgtctgggggcaaata-3’	Mouse/Primers (SYBR)
	Reverse: 5’-tcgttgagatgcttggggtc-3’	NM_007616.4
Fabp1	Forward: 5’- aaggcagtcgtcaagctgg-3’	Mouse/Primers (SYBR)
	Reverse: 5’-cattgagttcagtcacggactt-3’	NM_017399.5
Fabp4	Forward: 5’- agctggtggtggaatgtgtt-3’	Mouse/Primers (SYBR)
	Reverse: 5’-cttcctttggctcatgccct-3’	NM_024406.3
Plin3	Forward: 5’- tcatggcccacaatagcaaga-3’	Mouse/Primers (SYBR)

	Reverse: 5’-tgctagccggcctttaatc-3’	NM_025836.3
Plin5	Forward: 5’- ctcaactttcctgcccgtca-3’	Mouse/Primers (SYBR)
	Reverse: 5’-accggacattctgctgtgtg-3’	NM_001077348.1
Cpt1α	Forward: 5’- ctatgcgctactcgctgaagg-3’	Mouse/Primers (SYBR)
	Reverse: 5’-ggctttcgacccgagaaga-3’	NM_013495.2
Cpt1β	Forward: 5’- gacttccggcttagtcggg-3’	Mouse/Primers (SYBR)
	Reverse: 5’-gaataaggcgtttcttccagga-3’	NM_009948.2
Mttp	Forward: 5’- aatgcgggtcaacagagagg-3’	Mouse/Primers (SYBR)
	Reverse: 5’-ctggctcgttttcataggagtag-3’	NM_008642.3
Apob	Forward: 5’- gctcaactcaggttaccgtga-3’	Mouse/Primers (SYBR)
	Reverse: 5’-agggtgtactggcaagtttgg-3’	NM_009693.2
Gapdh	Proprietary to ABI	Mouse/Mm99999915_g1
Gapdh	Forward: 5’- acagcaacagggtggtggac-3’	Mouse/Primers (SYBR)
	Reverse: 5’- tttgagggtgcagcgaactt-3’	NM_017008.4

Table S8. Antibodies used in Western blot, co-immunoprecipitation, immunofluorescence and immunohistochemistry

Protein	Host	Company / Catalog	Antibody dilution
Gapdh	Rabbit	Proteintech, Chicago, IL/10494-1-AP	WB 1:3000
Na^+/K^+ATPase	Rabbit	Abcam, Cambridge, MA/ab76020	WB 1:10,000
Sema7a	Rabbit	Abcam, Cambridge, MA/ab23578	WB 1:1000, IHC1:50, IF:1:200, IP:2μg per sample
Integrin β1	Rabbit	Abcam, Cambridge, MA/ab183666	WB 1:1000
Plexin C1	Mouse	Santa Cruz, Dallas, CA/sc-390216	WB 1:1000
PKCα	Rabbit	Proteintech,Chicago, IL/21991-1-AP	WB 1:1000
p-PKCα(pT638)	Rabbit	Epitomics, Burlingme, CA/1195	WB 1:20000
Acc1	Rabbit	Bioworld Technology, St. Louis Park, MN/BS1378	WB 1:1000
Fasn	Rabbit	Cell Signaling, Beverly, MA/3180	WB 1:1000
Scd1	Rabbit	Cell Signaling, Beverly, MA/2794S	WB 1:1000, IF 1:200
Gpat	Rabbit	Proteintech,Chicago, IL/15401-1-AP	WB 1:1000
Lipin2	Rabbit	Abcam, Cambridge, MA/ab176347	WB 1:2000
Cd36	Rabbit	Abcam, Cambridge, MA/ab133625	WB 1:2000
Cd36	Mouse	Proteintech,Chicago, IL/66395-1-Ig	WB 1:1000
Fatp5	Rabbit	Abcam, Cambridge, MA/ab97884	WB 1:1000
Caveolin1	Rabbit	Cell Signaling, Beverly, MA/3267S	WB 1:1000
Plin3	Rabbit	Proteintech,Chicago, IL/10694-1-AP	WB 1:1000
Plin5	Rabbit	Proteintech,Chicago, IL/26951-1-AP	WB 1:2000
CPT1β	Rabbit	Abcam, Cambridge, MA/ab134988	WB 1:1000
Srebp1	Rabbit	Bioss Antibodies, Woburn, MA/bs-1402R	WB 1:1000
Chrebp	Rabbit	Novus Biologicals, Littleton, CO/NB400-135	WB 1:1000
Lxr	Rabbit	Proteintech,Chicago, IL/14351-1-AP	WB 1:1000
Lamin A	Rabbit	Abcam, Cambridge, MA/ab26300	WB 1:5000
Rabbit IgG	Rabbit	Proteintech,Chicago, IL/B900610	IP:2μg per sample, IHC1:50, IF:1:200

Suppl. References

1. Sun DQ, et al. PNPLA3 rs738409 is associated with renal glomerular and tubular injury in NAFLD patients with persistently normal ALT levels. *Liver Int*. 2020; 40(1):107-119.

2. Kleiner DE, et al. Nonalcoholic Steatohepatitis Clinical Research Network. Design and validation of a histological scoring system for nonalcoholic fatty liver disease. *Hepatology*. 2005;41(6):1313-1321.

3. Brunt EM, et al. Nonalcoholic steatohepatitis: a proposal for grading and staging the histological lesions. *Am J Gastroenterol*. 1999;94(9):2467-2474

4. Xu, Ziye, et al. Cold-induced lipid dynamics and transcriptional programs in white adipose tissue. *BMC Biology*. 2019, 17(1): 1-20.

5. Chai J, et al. Canalicular membrane MRP2/ABCC2 internalization is determined by Ezrin Thr567 phosphorylation in human obstructive cholestasis. *J Hepatol*. 2015; 63(6):1440-1448.
